# Environmental Pressure May Change the Composition Protein Disorder in Prokaryotes

**DOI:** 10.1371/journal.pone.0133990

**Published:** 2015-08-07

**Authors:** Esmeralda Vicedo, Avner Schlessinger, Burkhard Rost

**Affiliations:** 1 TUM, Department of Informatics, Bioinformatics & Computational Biology—i12, Boltzmannstr. 3, 85748 Garching, Munich, Germany; 2 TUM Graduate School of Information Science in Health (GSISH), Boltzmannstr. 11, 85748 Garching, Munich, Germany; 3 Icahn School of Medicine at Mount Sinai, Department of Pharmacology and Systems Therapeutics, One Gustave L. Levy Place, Box 1603, New York, New York, 10029, United States of America; 4 Institute of Advanced Study (TUM-IAS), Lichtenbergstr. 2a, 85748 Garching, Munich, Germany; 5 Institute for Food and Plant Sciences WZW Weihenstephan, Alte Akademie 8, Freising, Germany; Weizmann Institute of Science, ISRAEL

## Abstract

Many prokaryotic organisms have adapted to incredibly extreme habitats. The genomes of such extremophiles differ from their non-extremophile relatives. For example, some proteins in thermophiles sustain high temperatures by being more compact than homologs in non-extremophiles. Conversely, some proteins have increased volumes to compensate for freezing effects in psychrophiles that survive in the cold. Here, we revealed that some differences in organisms surviving in extreme habitats correlate with a simple single feature, namely the fraction of proteins predicted to have long disordered regions. We predicted disorder with different methods for 46 completely sequenced organisms from diverse habitats and found a correlation between protein disorder and the extremity of the environment. More specifically, the overall percentage of proteins with long disordered regions tended to be more similar between organisms of similar habitats than between organisms of similar taxonomy. For example, predictions tended to detect substantially more proteins with long disordered regions in prokaryotic halophiles (survive high salt) than in their taxonomic neighbors. Another peculiar environment is that of high radiation survived, e.g. by *Deinococcus radiodurans*. The relatively high fraction of disorder predicted in this extremophile might provide a shield against mutations. Although our analysis fails to establish causation, the observed correlation between such a simplistic, coarse-grained, microscopic molecular feature (disorder content) and a macroscopic variable (habitat) remains stunning.

## Introduction

### Disordered regions might contribute to complexity of an organism

We refer to disordered regions as those long stretches of consecutive residues in proteins that do not adopt well-defined three-dimensional (3D) structures in isolation [1]. Proteins with long disordered regions encompass some unique biophysical characteristics which allow them to bind to several different partners, often at different times and under different cellular conditions [2]. Typically regions with at least 30 consecutive residues predicted as disordered are considered as “long”. Computational predictions have noted an overabundance of disordered regions in protein interaction hubs [3–7] and in transcriptional master regulators [8, 9]. Proteins with disordered regions appear to be particularly abundant in processes such as transcription, translation, signal transduction, and macromolecular transport through the nuclear pore complex [4, 10, 11]. All these observations support the to some degree oversimplified view of disordered regions as building blocks for system complexity [1]. On the level of kingdoms: 10–20% of all proteins from prokaryotes have at least one long disordered region, while 20–50% of all eukaryotic proteins do [1, 12, 13]. Recent comparative proteomics studies have strengthened the link between disorder and organism complexity, e.g. disordered regions in ancient branching eukaryotes appear to differ from those in other eukaryotes [14–16].

### Comparative proteomics reveals new evolutionary links

How does the complexity of an organism evolve? Do humans share a minimal set of genes with bacteria and have all others evolved for non-bacteria specific functions [17]? These two questions have been pursued by many comparative genomics studies [18] for many years; the final explanation is still being sought after. One approach to comparing genomes is to focus on characteristics of proteins. For example, combining analysis of sequence, structure, expression and evolutionary relationship information of multiple protein data sets from yeast, mouse and human, evidence could be found about the relationships between divergence in the length of disordered regions and changes in the protein functions [19]. A modification of the length of disordered regions in paralog proteins might provide a simple evolutionary mechanism for protein degradation rates. As many of these affected paralogs were participating in protein signaling pathways, the cellular function and phenotype of the cells would also be influenced by these changes [20–22]. It is also a well-known fact that intertwined helices (coiled-coils) are highly over represented in eukaryotes [23]. Helices might constitute excellent evolutionary building blocks as they can form exclusively from local internal molecular interactions [24]. Through the application of prediction methods, we can integrate this useful information to compare structural features across species for entire proteomes [11, 17, 23, 25–28]. In our study we focus on the study of simple, average features from predictions that can be obtained for entire organisms.

### How do prokaryotic proteins adapt to the extreme?

It appears intuitive to assume that increasing the internal inter-residue bonds in a protein raises its stability at high temperature. Several studies have, indeed, reported correlations between thermal stability and features such as a high contact density and unusual numbers of hydrogen bonds [29, 30]. A difference in the average amino acid composition was found when considering in more detail the amino acid composition, the sequence of proteins from thermophiles and those of mesophiles [31]. Protein structures from thermophiles such as *Pyrococcus horiskoshi* OT3 have been reported to contain more intra-helical salt bridges than their homologues in mesophiles [32]. These salt bridges are an important factor stabilizing thermophilic proteins [30]. All these findings suggest that diverse factors determine thermostability [33]. Psychrophiles live in the extreme cold. Recent studies have suggested that proteins from psychrophiles increase their flexibility and accessibility and might thereby hinder freezing [34]. Proteins from *halobacteria* (salty habitats) also exhibit unique characteristics such as low hydrophobicity, excess of acidic residues, depletion of cysteine residues and reduced propensities for helix formation [35]. All these observations induced us to hypothesize that protein disorder might somehow correlate with habitat.

Assuming that protein disorder plays a marginal role in prokaryotes, most studies have focused on eukaryotes. Here, we zoomed into protein disorder abundance across prokaryotes. Specifically, our first question was whether the overall percentage of proteins with long regions of protein disorder is associated with organism habitat, or alternatively, with taxonomic distance. Put differently: are two proteomes more similar in their disorder content when they are related by evolution or when they live in similar habitats? We predicted disorder through several *in silico* methods applied to about 46 organisms that thrive in different habitats. Overall, we claim to have established a stronger correlation between disorder and habitat than between disorder and taxonomy for the same control set. Furthermore, our results appeared more compatible with the idea of “gradual adaptation” than with that of “gradual leap”, i.e. disorder regions were added to many proteins, rather than introducing a few new, organism-specific proteins with disordered regions.

## Methods

### Data

The UniProt database [36] provided the complete proteome sequence data at the basis of our study. We removed all duplicates (giving priority to longer proteins) and applied no other filtering. Our analysis considered 46 organisms with a total of 225,550 proteins (S1 Table). The organisms sampled the most extreme habitats and their closest completely sequenced relatives. We also included a few selected eukaryotes for comparison.

Most information used to classify organisms was taken from GOLD (Genomes Online database version 2011-09-23 [37]). We avoided pathogens, parasites, and other biotic relationships to build a “simplified” subset of organisms. We classified into the following types of environment (S1 Table) [38–40]: thermophiles (optimal growth at 45–80° C), hyperthermophiles (optima >80° C), pychrophiles (optimal growth at about 15° C, a maximal temperature for growth at about 20° C, and a minimal temperature for growth at 0° C or below), psychrotolerants (organisms that are not considered as pyschrophile but have the capability for growth at 0° C or close to 0° C), halophiles (optimal growth in salt solutions, i.e. from 25% NaCl up to saturation), alkaliphiles (optimal growth around pH>8), mesophiles (including bacteria and archaea from “normal” environments). Eukaryotes were considered as a different group as they have a different content of disorder [1].

### Disorder prediction

We used prediction methods that were developed based on different concepts and capture different “flavors” of protein disorder [6, 41, 42]. Therefore when analyzing the predicted amount of disordered proteins in an organism, it is possible to obtain distinct values depending on the predictor. IUPred uses pairwise statistical potentials of residue contacts [43, 44] and has been presented as an unbiased and robust predictor even for organisms living in extreme habitats [45, 46]; Meta-Disorder (MD) [42] and NORSnet [6] are neural network-based methods that use evolutionary information and other predicted features. MD combines several original prediction methods including NORSnet, with evolutionary profiles and sequence features that correlate with protein disorder such as predicted solvent accessibility and protein flexibility. NORSnet is focused on the identification of long disordered loops (no regular secondary structure, namely “loopy disorders”); it is optimized without using any experimental data on disorder. Disordered regions that are not predicted to be “loopy” are considered as “regular” disordered regions.

There are many alternatives how to compile overall averages for protein. We analyzed almost the entire resulting data avalanche and found most alternatives to be redundant. Therefore, we focused on as few alternatives as possible; we included different views only if they provided important additional information. In particular, we considered three thresholds to define “long disorder”: **%long30**, is the percentage of proteins with at least one region of ≥30 consecutive residues predicted as disordered (**%long50** and **%long80** were the same with length thresholds at ≥50 and ≥80, respectively). We also investigated another extreme concept, in particular that of a protein that is **completely disordered** (S1 Fig): if a protein had no single region that we could perceive as a “nucleation site” for adopting regular structure, we considered this protein as completely disordered. Operationally, we first removed any prediction of disorder that spanned over fewer than five residues; next we searched any region without predicted disorder over 30 consecutive residues. If we found no such region, and if we also found at least one region with ≥30 consecutive residues predicted as disordered, we considered the protein to be completely disordered. All thresholds were tested with three prediction methods, concretely MD, NORSnet and IUPred. To simplify comparisons between these three, we replaced their raw scores by Z-scores, i.e. gave the score as a deviation from the average in units of one standard deviation:

$$\text{z}\left({}_{\text{o},\;\text{M}}\right)=\frac{\text{raw}\left({}_{\text{o},\;\text{M}}\right)-<\text{raw}>\left({}_{\text{all organisms},\;\text{M}}\right)}{\sigma \left({}_{\text{all organisms},\;M}\right)}$$ (Eq 1)

where *z(*_*o*,*M*_ *)* is the Z-score for a particular method M and organism o, raw(_o,M_) is the raw score of prediction method M for organism *o* (e.g. the percentage of proteins with at least one region of long disorder in *o*), < raw > _(allorganisms¸M)_ is the average over the raw scores for method M over all organisms, and σ_(allorganisms¸M)_ is the standard deviation for the distribution of the raw scores predicted for all organisms by method M. Positive Z-scores imply a disorder content higher than the mean, negative scores lower than the mean. We compiled averages and standard deviations over a set of 1,613 complete prokaryotic proteomes from UniProt (with almost 90% of the sequences predicted by the three predictors) in order to have a Z-score calculated independently of the samples selected and to give more information compared to the total of the 1,613 organisms. Eukaryotes were not included in this computation due to the difference in disorder content [13]; they were considered separately for the analysis. The calculated means (*ave*) and standard deviations (*sd*) for **“%long30**” were: MD_ave_ = 14.6%, MD_sd_ = 4.2%; NORsnet_ave_ = 2.5%, NORsnet_sd_ = 2.0%; IUPred_ave_ = 7.5% and IUPred_sd_ = 5.5% (for other approaches see S3–S5 Tables).

### Tree of life

We constructed and visualized the tree of life using the interactive *Tree of Life* (ITOL) webserver [47, 48]. Taxonomic identifiers for the organisms were taken from UniProt and uploaded into the NCBI taxonomy browser [49, 50] to automatically generate a phylogenetic tree in *phylip* format [51]. The resulting tree was visualized using the “Multi-value Bar Chart” a circular mode of ITOL.

### Defining homology

In order to identify phylogenetic relations such as the homology of proteins between the thermophile *Pyrococcus horikoshii OT3* [52] and the model organism for the study of life in permanently cold environments *Colwellia psychrerythraea 34H* [53], we applied the following *ad hoc* procedure: We blasted [54] all protein sequences from one organism against all from the other. For each resulting alignment we calculated the HSSP-value (HVAL) [55–57], which measures sequence similarity by combining alignment length and percentage of pairwise sequence identity. For instance, HVAL = 0 corresponds to about 22% pairwise sequence identity for alignments over 250 residues. As a result of our procedure, proteins can have multiple homologues. Due to technical concerns, we grouped all relations found avoiding the problem in the distinction between paralogs and orthologs [58, 59].

### Statistical tests

In addition to the similarity between proteins from two organisms, we also assessed the statistical significance of disorder content comparisons between organisms with similar habitat (S1 Table) and with similar phylogeny (S14 Table). In particular, we applied the Kruskal-Wallis test (H-test) [60, 61], the Wilcoxon signed-rank test [62–65] and the Brown–Forsythe Levene’s test (also known as Levene’s test) [66, 67] (S2 Fig). The non-parametric Kruskal-Wallis test compares the shape of the distributions between two or more unmatched groups for nominal variables of small and unequal sample size and determines whether the distributions of the groups are identical (null hypothesis) [60, 62, 63]. The pairwise Wilcoxon signed-rank test is a nonparametric test for matched or paired data to assess whether the differences of the median between pairs of observations is zero [62–65]. The Levene’s test is a non-parametric test that also works for non-Normal (non-Gaussian) distributions; it determines if all variances between groups are zero (null hypothesis, α = 0.05) [66, 67]. For all the statistical tests, we used the median for each group either habitat or phyla, calculated from the protein disorder content of the organisms belonging to this group.

The Kruskal-Wallis test does not assume a normal distribution for the data but homoscedasticity (not significant differences between the group variances) [60, 61] therefore first, we performed the Levene’s test of equality of variances (S2 Fig). If the Levene’s test failed for the overall comparison across the groups, then we performed pairwise comparisons between the groups (S2 Fig). For those groups for which the null hypothesis (equal variances) is accepted, a pairwise Wilcoxon signed-rank test will be applied as alternative to the Kruskal-Wallis Test (null hypothesis: groups have equal distribution; α = 0.05; S2 Fig). The groups rejecting the null hypothesis and therefore presenting a significant difference of disorder content distribution were all marked with asterisks (P< 0.05 with * and P <0.005 with **). The pairwise Wilcoxon signed-rank test was also applied when the Kruskal-Wallis test failed for the overall comparison test (accept alternative hypothesis, i.e. at least one group in the population for which the distribution of disordered protein contents differs from the others) and after the null hypothesis of the pairwise homogeneity Levene’s test was accepted (S2 Fig). Furthermore, habitat is a complex reality defined by a variety of ambient conditions and organism properties which have to be studied separately. For that we also analyzed, some of the general properties of the organisms (metadata) included by the GOLD database [37]. For the statistical analysis groups containing less than two samples were not considered. All analyses were performed using the R software (statistical packages car and stats) [66, 68].

## Results & Discussion

### Salty habitats are dominated by high disorder

**Fig 1:**
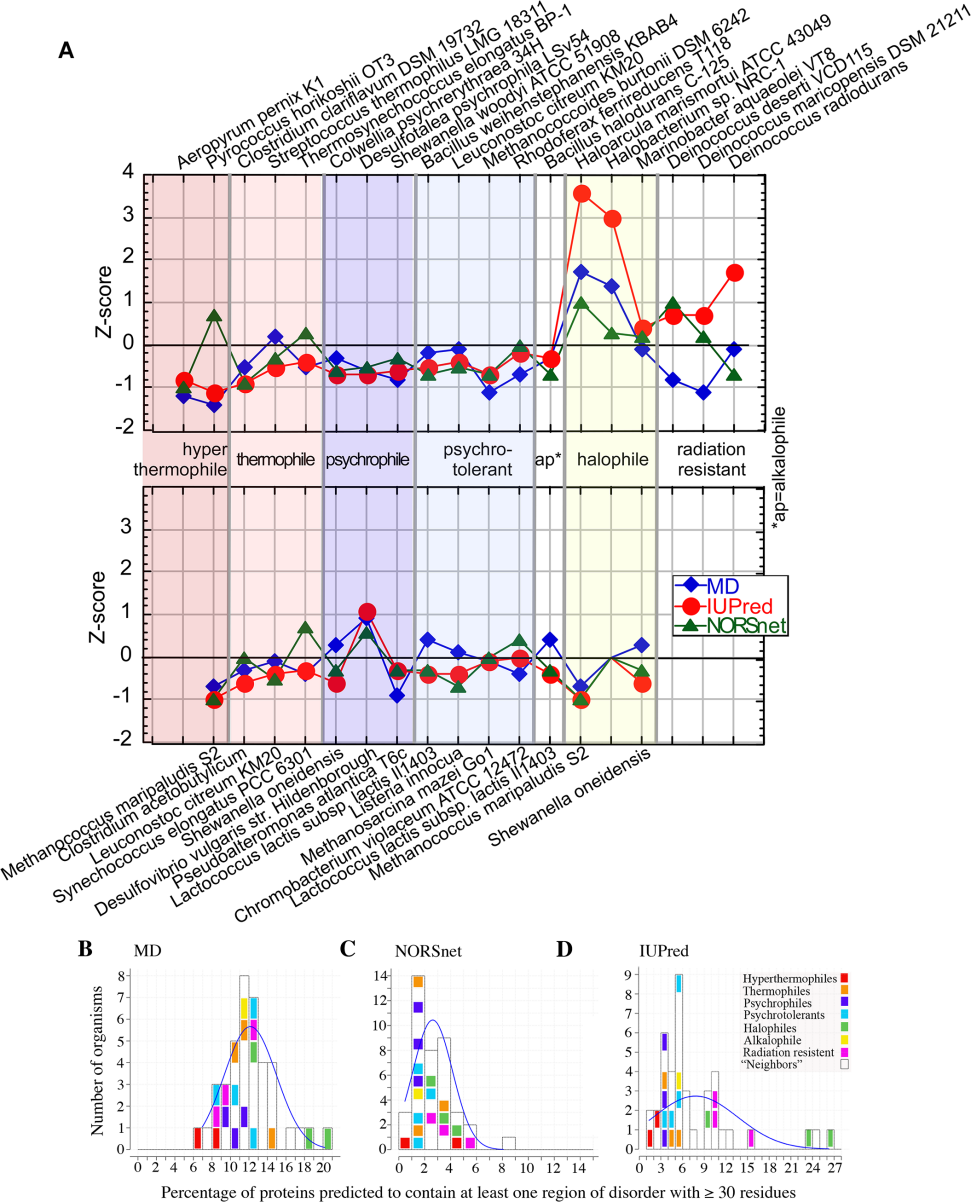
Distribution of disorder content in different organisms. Fractions of proteins with long regions of disorder (here ≥30 consecutive residues) were predicted by three prediction methods (MD, NORSnet and IUPred). **(A)** The raw values are standardized using the Z-scores (Eq 1; mean and standard deviation σ from a 1613 prokaryotes calculated for each method; positive: higher than the mean; negative: below the mean; integers +/- N imply N*σ above/below the mean). The top panel shows the extremophiles; the lower panel shows the closest phylogenetic relative for each extremophile in the top panel (for relatives discussed in the text and left out for clarity from the figure, for all studied organisms S3 Fig). The archaeal halophiles *Haloarcula marismortui ATCC 43049* and *Halobacterium sp*. *NRC-1* were predicted with the highest content of proteins with long disorder. Conversely, the archaeal thermophile *Aeropyrum pernix K1* was one of the organisms predicted with the lowest disorder. The taxonomic neighbors section compares the disorder predicted for the closest relatives of the extremophiles. **(B-D)** Mapping of disorder protein content predictions for all organisms for each prediction method (B: MD [42], C: NORSnet [6], and D: IUPred Clearly, all three methods put the thermophiles on the left (less disorder), while the halophiles appear on the right (high disorder). The blue curves are Gaussian fits based on the mean and σ of our data.

Halophiles thrive in salt-saturated habitats. The percentages of proteins predicted with long disorder in the two halophilic archaea *Halobacterium sp*. *NRC-1* [69] and *Haloarcula marismortui ATCC 43049* [70] both reached levels around 20–28% (percentage of proteins with at least one region with >30 consecutive residues predicted to be disordered by MD and IUPred). This was much higher than average (Z-scores Fig 1A, note Z-score = 0 implies ‘like average’, +1/-1: imply values one standard deviation above/below average) and much higher than the values for their closest taxonomic relative *Methanococcus maripaludis* S2 [71] (Z-scores<-0.5 Fig 1A) that does not survive in high salt. The same tendency was observed for the other methods and thresholds (S7 and S8 Tables).

The difference in disorder abundance between the halophilic bacterium *Marinobacter aquaeolei VT8* [1] (Z-score around 0, Fig 1A) and its taxonomic relative *Pseudoalteromonas atlantica T6c* [72] (Z-score around -0.5, Fig 1A) was not as pronounced as for the archaea, but it confirmed the “high disorder in salt” trend for bacteria. The difference in disorder between halophile and relative was slightly higher for longer disorder (S8 Table and S4 and S5 Figs). When considering the percentage of proteins considered as completely disordered (S1 Fig), the difference increased (S2 Table vs. S9 Table). The difference was the same in relative terms for a method that detects only long loops (no regular secondary structure, such as NORSnet) as disorder, although the content for that method dropped significantly (NORSnet in Fig 1A). These observations across different phyla might suggest the increase in disordered regions as one means for prokaryotes to cope with high salt-conditions. This result has been reported before [45, 73]. New here is the relation between phylogeny (closest relatives) and extremity of habitat (high salt).

### Is disorder slightly lower in hot habitats?

Organisms surviving in extreme heat have been reported to have rather low levels of disorder content before ([45]). The group of Peter Tompa–[45]—also reported a low content of disorder in organisms surviving the cold and put these results into perspective of evolutionary relatives. Here, we repeated their analysis in a slightly wider context, largely confirming their findings.

The hyperthermophile *Pyroccocus* [74] might be the most studied organism living in very high temperature (close to 100°C) and greater sea depth than other archaea (pressures reaching 200 bar, i.e. ~200 times what we live in). At least for two of the methods we analyzed, *Pyroccocus horikoshii OT3* [75] was predicted with very little long disorder (>30 residues, Fig 1A: <-1, i.e. over one standard deviation below average). The closest relative, *Methanococcus maripaludis S2*, was predicted with similar low disorder (Z-score around -1 Fig 1A). The optimal growth temperature for *Methanococcus maripaludis* is 35–40°C, i.e. “normal”, and it is isolated from salt marsh sediments. Following our simple logic, we expect two reasons for *Methanococcus* to have higher disorder than *Pyroccocus*: salt (higher disorder) and less heat (higher disorder). For our method predicting loopy disorder, the trend was even inversed. We failed to explain why we did not observe this.

*Aeropyrum pernix K1* (isolated from sulfur-rich under-sea vents in Japan) [76–78] is another hyperthermophile archeae. Like *Pyroccocus*, *Aeropyrum* was predicted with very little disorder (Z-score ~-1, Fig 1A). This was similar to other hyperthermophiles that we sampled. Analogous to the halophiles, the “loopy” disorder predicted by NORSnet, was even lower for these hyperthermophiles than the “regular” disorder. While we might jump into suspecting that shortening connections between regular secondary structure segments (helices and strands) might protect against heat and high salt, we should speculate with care because this seems incompatible with the prediction of “loopy disorder” for *Pyroccocus* (Fig 1A).

### Disorder seems not higher in cold habitats

*Colwellia psychrerythraea 34H* [53] is considered as an obligate psychrophile marine bacterium, i.e. it needs very low temperatures (-1°C to +10°C) to grow; it can support high pressures in the deep sea. Its predicted disorder was below average (Z-score about -0.5, Fig 1A). *Leuconostoc citreum KM20* [79] is considered to be a psychrotolerant antimicrobial producer (used for fermentation of kimchi). It grows optimally at 30°C, but can also be cultivated at significantly higher temperatures. Its predicted disorder was also below average (Z-score about -0.5; Fig 1).

A recent study provided experimental evidence that proteins with long disordered regions can be more stable in cold temperatures than globular proteins [80]. Our predictions for entire genomes seemed incompatible with the concept that such a solution would be imprinted upon the entire proteome. If anything, our analysis of psychrophiles confirmed previous findings that organisms in cold habitats have less disorder than average ([45]).

### Is high disorder protecting from radiation?

*Deinoccocus radiodurans R1* [81, 82] is often jokingly referred to as “Conan the bacterium” because it tolerates many extreme conditions including radiation, cold, dehydration, heat and high acidity. We predicted a high abundance of protein disorder in this bacterium (Z-score between 0 and 2: Fig 1A). We only found two taxonomic neighbors of *Deinoccocus radiodurans*: *Deinococcus deserti* and *Deinoccus maricopensis*. Both also sustain high radiation and live in the dry: *Deinococcus deserti* and *Deinoccus maricopensis* (Z-scores >0 for IUPred, Fig 1A). The ‘high radiation’ habitat was particularly inconsistent between the three prediction methods: e.g. MD predicted the opposite (Fig 1A). Inconsistency between prediction methods might suggest taking the correlation ‘high radiation—high disorder’ with a grain of salt. Conversely, we might argue for the opposite: IUPred, MD, and NORSnet rely on partially orthogonal information. This independence might imply that some reality might be discovered by only one of the methods, namely the one better able to capture that reality.

### No clear trends for other disorder outliers

Finally, we analyzed the disorder abundance in prokaryotes that live in other extreme habitats including high pH (*Bacillus halodurans* [83], disorder below average, Fig 1A) and changing environments (*Shewanella oenidenses* [84], disorder around average, Fig 1A). However, so far we failed to notice significant trends (Fig 1A). Moreover, we failed to explain why some mesophiles were outliers (higher or lower content of disordered proteins). For example, *Caulobacter vibrioides* (also known as *Caulobacter crescentus*) [56] was predicted with high disorder (Z-score one standard deviation above average, Fig 1A) without any apparent reason. *Caulobacter* secretes Nature’s strongest glue [85, 86]. This might point to another important role for high content of disorder. *Streptomyces coelicor* was also predicted with higher than average disorder (Z-score >1, Fig 1A); this might be explained by its complex life cycle and production of antibiotics (their products are pharmaceutically used as anti-tumors agents, immunosuppressants and antibiotics).

*Ruegeria pomeroyi DSS-3* [87] (originally classified as *Silicibacter pomeroyi* [88]) was predicted with very low disorder (Z-score about -1, Fig 1A). Its taxonomic neighbor, *Rhodobacter sphaeroides 2*.*4*.*1*, was predicted at above average disorder (Z-score>0, Fig 1A). *Ruegeria* was isolated from seawater off the US-Southeast coast; it lives at 10–40°C and grows with and without carbon monoxide (CO) as carbon source. We cannot explain the low protein disorder content predicted for *Ruegeria*.

### Detailed analysis of corresponding homologues brings new insights

We calculated disorder abundance in organism specific and homologues of two model organisms representing two extreme temperature environments, using various thresholds in terms of sequence similarity to define homology (Table 1). The aim was to analyze whether the aligned region of the corresponding homologues from two opposing extremophiles (heat/cold) includes the disordered region or not. In particular, we compared the homologues between the low-temperature/low-disorder psychrophile *Colwellia psychrerythraea 34H* and the high-temperature hyperthermophile *Pyrococcus horikoshii OT3*.

**Table 1:** Protein disorder overlap between related proteins in opposite extremophiles.

HVAL ^*a*^	*Colwellia psychrerythraea* 34H (freeze)		*Pyrococcus horikoshii* OT3 (heat)	
	*related* ^*b*^	*related+disordered* ^*c*^	*related* ^*b*^	*related+disordered* ^*c*^
-20	75.5 ±0.2	9.5 ± 0.1	66.9 ± 0.1	5.53 ± 0.06
-10	56.4 ±0.2	6.8 ± 0.1	55.7 ± 0.2	5.04 ± 0.08
0	24.0 ±0.1	4.9 ± 0.2	30.9 ± 0.1	2.7 ± 0.1
10	5.5 ±0.1	2.6 ± 0.2	9.7 ± 0.1	0.51 ± 0.06
20	0.6 ±0.02	0.07 ± 0.02	1.28 ± 0.03	0
30	0.04±0.01	0	0.20 ± 0.01	0

*a* **HVAL** measures sequence similarity as the distance from the HSSP-curve [55, 89]; e.g. HVAL = 0 implies 20% pairwise sequence identity (PIDE) for >250 aligned residues [57] (or 20+N% PIDE at HVAL = N).

*b* **related** gives the percentage of proteins in one organism (CP: *Colwellia psychrerythraea* 34H or PH: *Pyrococcus horikoshii*) that have corresponding homologs in the other (PH or CP) at the given HVAL ^***a***^ (totals: CP = 4423 and PH = 1573). For instance, 24% of all 4423 CT proteins have a match in one of the 1573 PH proteins at HVAL≥0, while almost 31% of the PH proteins have a homolog in CP at this level of sequence relation. One standard error is marked as ‘±stderr’.

*c* **related+disordered** gives the percentage of proteins in one organism (CP or PH) that are related ^***b***^ and have at least one disordered region (>30 residues, prediction by MD; other methods and thresholds in SOM) in the other (PH or CP) at the given HVAL ^***a***^. Overall MD predicts 12% of all *Colwellia psychrerythraea* 34H and 8% of all *Pyrococcus horikoshii* OT3 proteins to have at least one long disordered region (Table 1; cold = high disorder, heat = low). These numbers imply that the proteins shared between the two extremophiles from opposite ends of the temperature spectrum are depleted in disorder with respect to the entire proteome. For instance only 4.9% are related and disordered from the CP perspective at HVAL≥0 as opposed to 12% for all proteins. The more similar the homologs the more the related proteins were selected to not contain disorder. One standard error is marked as ‘±stderr’.

At pairwise protein similarity levels of HVAL≥10 (corresponding to about 30% pairwise sequence identity over 250 aligned residues), seven of the homologs with disorder in *Colwellia* (cold) had no disorder in *Pyrococcus* (heat; S9 Table); the number for the flipside control was: one protein with disorder in *Pyrococcus* and not in *Colwellia*.

Several studies investigating the effect of temperature on enzymes–which are disorder depleted as a class of proteins—showed that proteins from extremophiles (both cold and hot) adopt similar structures as their mesophilic orthologs, but use different amino acids to compensate for temperature effects [30, 31, 34]. Our analysis confirms this trend (S6A Fig), the particular choice of amino acids in whole proteomes of hyperthermophile (S6A Fig: red) and thermophile (S6A Fig: blue) were slightly different compared to that for psychrophile (S6A Fig: green) and psychrotolerant (S6A Fig: purple) organisms. However, the differences were significant at best for some particular amino acids. The strongest signal was for negatively charged amino acids such as glutamic acid (E, S6A Fig), that occurred more in heat than in cold. The situation was, however, almost inversed for the negatively charged and slightly less acidic aspartic acid (D, S6A Fig). Glutamic acid might be abundant in heat to favor electrostatic interactions in these proteins and thereby increase their stability [90]. The only other amino acid occurring more often in thermophiles and hyperthermophiles was tyrosine (Y, S6A Fig). On the other hand, the hydrophobic methionine (M, S6A Fig) was over-represented in both psychrophiles and psychrotolerants. When grouping all amino acids in two classes (hydrophobic/not) using different hydrophobicity scales (Eisenberg and Weiss [91], Kyte-Doolittle [92], and Janin [93]), we could confirm the observation [34] that psychrophiles have less hydrophobic residues than hyperthermophiles (but not less than thermophiles): the differences we observed between the antipodes (cold/heat, S6B Fig) were insignificant (Z-score between -0.05 and -0.1- for the psychrophiles vs. 0.04–0.2 for the hyperthermophiles).

Let us nevertheless assume that our findings had established the amino acid differences to be significant so that organisms could adapt to opposite temperature scales by altering the amino acid composition in all proteins. If true, the proteins that are shared between different extremophiles would be aligned to each other independently of their disordered regions. If these observations were always true, all seven disordered regions from *Colwellia* would likely fall within the aligned regions from *Pyroccocus*. The discrepancy between the expected 32 disordered proteins and the observed 7 (S12 Table) could be explained by the fact that proteins from thermophilic organisms might “tighten the loops” [30] to increase thermostability, and psychrophilic proteins might “loosen the loops”, i.e. might use more flexible loops to compensate for freezing effects. This could explain the long gaps in the alignments between the two homologous proteins that far exceed those needed to align each of them to its mesophilic relative. An alternative explanation is that these unaligned, disordered regions from *Colwellia* function as antifreeze proteins, which are unique to psychrophiles, and are capable of binding ice crystals using a large surface, thereby lowering the temperature, or changing the physico-chemical surroundings of the organism [34].

Overall, it seems likely that the difference in disorder between *Colwellia* and *Pyroccocus* on opposite sides of a tremendous temperature spectrum largely originated from homologous proteins that kept their overall shape with some modifications to adapt to extreme climates. These modifications may include shorter loops, less surface area and more compact proteins in thermophiles, and exceptionally flexible proteins in psychrophiles. Our comparison between the two opposite (cold/heat) extremophiles suggested that overall the total disorder composition was affected by many small rather than by a few big changes.

### Disorder differs more between habitats than between phyla

**Fig 2:**
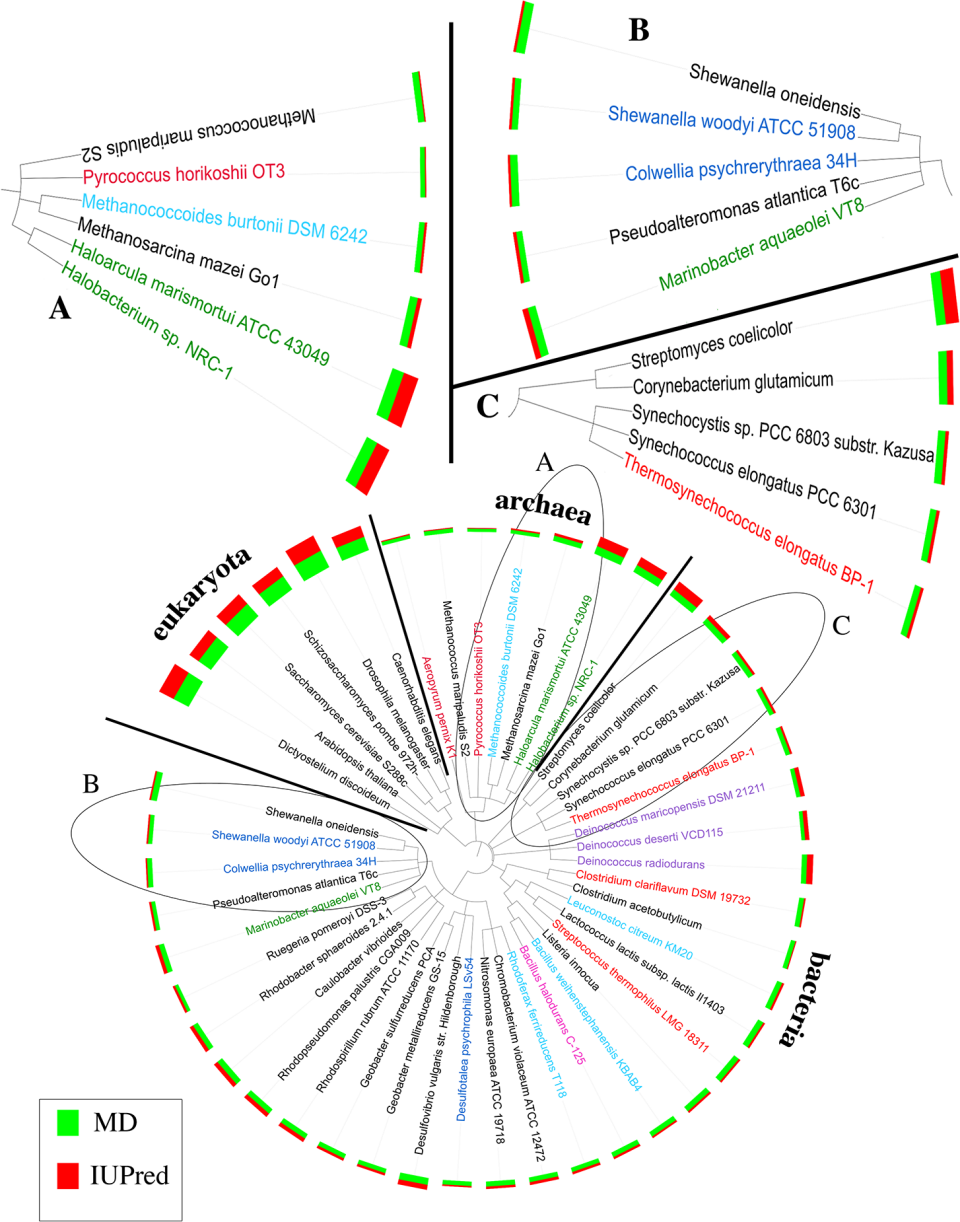
Protein disorder content differs for habitat, not for phyla. We represent the protein disorder content for the organisms in similar habitats (left panel) and those in the same phyla (right panel). The y-axes give the percentage of proteins with at least one region of ≥30 consecutive residues predicted as disordered by MD (A), NORSnet (B) and IUPred (C). The x-axis on the left side marks the different environmental groups (S2 Table); on the right side marks the studied phylogenetic groups (S14 Table). The groups which are significant for a paired Wilcoxon Test are marked with * (P<0.05) or ** (P<0.005).

Through the application of the Kruskal-Wallis and the paired Wilcoxon-Test, we found that the habitat groups presented different distribution of disordered content for MD (P<0.05; S15 Table and Fig 2A) and IUPred predictions (P<0.05, P<0.005; S17 Table and Fig 2B) and for all thresholds (%long30, %long50 and %long80; Fig 2 and S7 and S8 Figs). Conversely, the phyla groups largely did not differ in any statistically significant way (Fig 2; S15–S17 Tables). Exceptions were differences in protein disorder content between the groups for NORSnet (“loopy” disorder) for all thresholds, for MD only for the middle long disordered proteins (%long50 and only for one pair of the groups in %long30; S15 Table and Fig 2A.) and for IUPred for the proteins containing long disordered regions (%long80; S15–S17 Tables and S8C Fig). Thus, the “loopy” disorder appeared more conserved than other disordered regions [94]. But why were disordered regions longer than 80 consecutives residues affected? While we lack sound explanations, we observe that other studies support the opposite [20, 95–98]. When analyzing the completely disordered proteins we found that both, phyla and habitat have an influence on the disorder content distribution for the IUPred and NORSnet predictions but only for disordered regions with at least 50 consecutives disordered residues (%long50 and %long80; S18 Table and S7 and S8 Figs). All those observations were confirmed when considering Z-scores (S19–S22 Tables).

The habitat is a complex reality defined by a variety of factors such as temperature, pH, energy source and metabolism (S14 Table). We tried to analyze these factors as separately as possible and in doing so we also found a significant difference in disorder content between the organisms grouped by temperature (high temperature–low disorder; S15, S18 and S22 Tables) and by oxygen requirement (an aerobic lifestyle implied higher disorder [99, 100]; S15, S17–S19, S21 and S22 Tables). However, for the other factors (metabolism, energy source, cell shape, S15–S22 Tables) we did not observe a significant influence on disorder (content of proteins with long disordered regions). Finally, we could only suggest that in general the protein disorder abundance in proteomes is more related to environment than to phylogeny but this might be the opposite for “loopy” disorder.

### Null hypothesis that disorder similar between habitats clearly rejected

**Fig 3:**
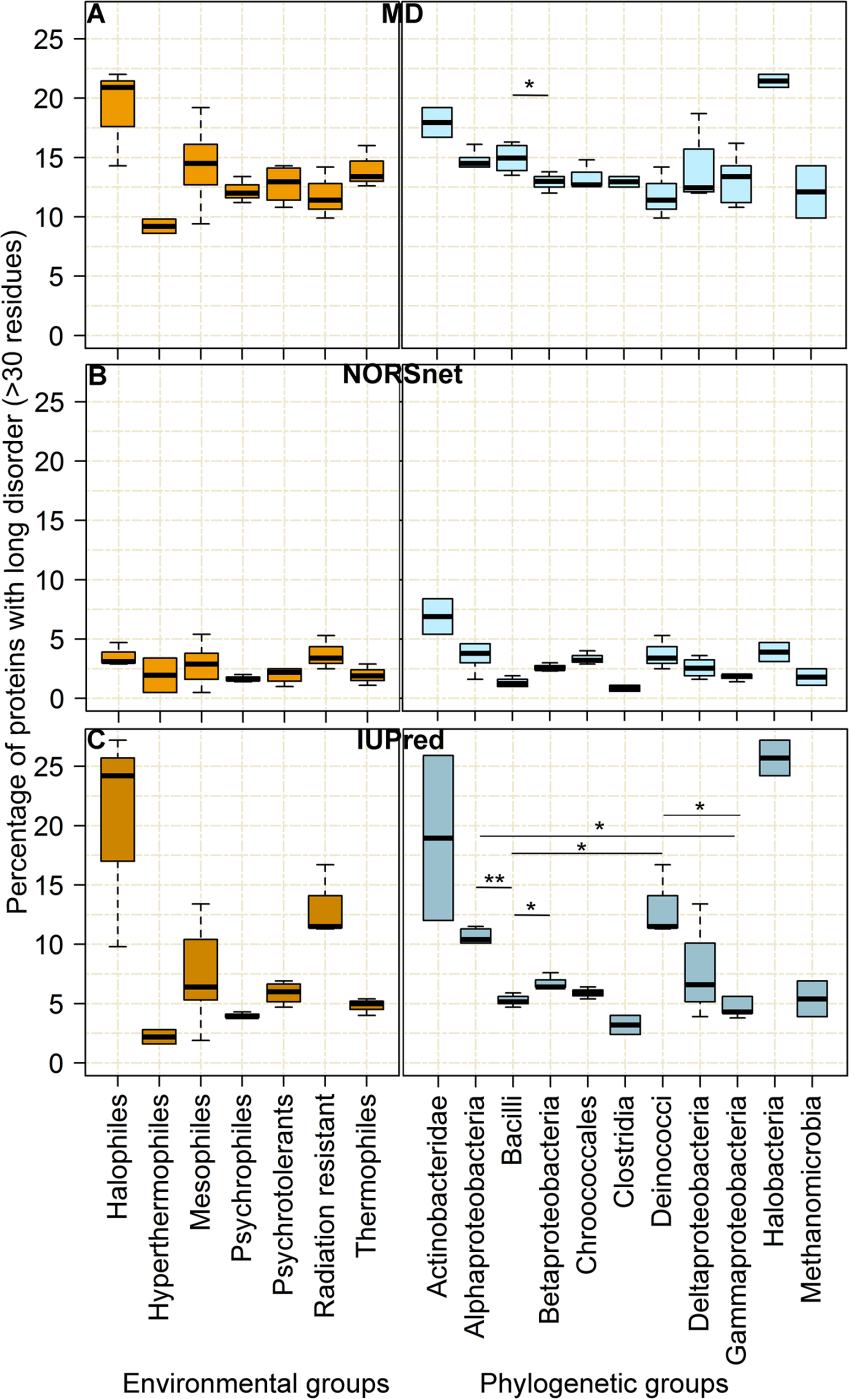
Protein disorder linked to habitat more than to phylogeny. The fractions of proteins with long disordered regions are predicted by two disorder predictor methods (MD in green bars and IUPred in red bars). Eukaryotes are predicted with substantially more disorder than prokaryotes. Within the kingdoms predictions vary greatly: organisms in similar habitats tend to resemble each other in terms of disorder more than they resemble their closest phylogenetic relatives. (A) Hyperthermophilic archaea (dark red) are more ordered than their phylogenetic neighbors; halophilic archaea are more disordered (green). (B) Halophilic bacteria also appear more disordered than their relatives. (C) The bacterial thermophile (red) also has less disorder than its relatives. Other extreme organisms included: psychrophile (blue), psychrotolerant (light blue), radiation resistant (purple) and alkalophile (pink). We could also find organisms with relative high/low disorder content explainable separately.

Protein disorder is much more abundant in eukaryotes than in prokaryotes ([10, 101]). Nevertheless, there are substantial differences between prokaryotes (Fig 3) that appeared to correlate more between habitats than between phyla, i.e. proteins from similar habitats appeared more similar in terms of the percentage of proteins with long disordered regions than proteins with similar phylogeny (Figs 1–3). Although we reported some examples for strong correlation between habitat and disorder, we also came across many examples of organisms for which our simple hypothesis predicted the opposite of what we observed. For instance, the hyperthermophile *Pyroccocus horikoshii* was predicted with below-average disorder while its closest relative *Methanococcus maripaludis S2* was predicted with similar low disorder although it cannot survive in the heat and survives high salt which we showed to correlate with high disorder. Another conundrum originated from the detailed comparison between two organisms at opposite ends of the temperature extremity: the low-temperature/low-disorder psychrophile *Colwellia psychrerythraea 34H* and the high-temperature hyperthermophile *Pyrococcus horikoshii OT3*. The detailed comparison of corresponding related proteins (‘orthologs’) provided evidence that longer loops and more disorder might help to survive in the extreme cold. On the level of entire organisms we observed the opposite (and thereby confirmed previous results ([45]). May be others will bring clarity to the confusion we find in the data. While our data might not suffice to clearly prove the correlations, the data is clear enough to reject the null hypothesis (disorder not correlated between habitats). In other words, there is a signal but it might remain hidden because it might be overshadowed by other constraints for survival.

What if the signal that we report were caused by mistakes in the method? We might suspect that prediction methods have not been developed for the type of organisms for which we apply these methods here. There is little evidence for the validity of this concern. For instance, secondary structure prediction methods developed over 22 years ago ([102]) continue to correctly capture the situation for very different proteins from very different environments than had been anticipated to exist 20 years ago (disorder just being one case in point–[10]). Similarly, none of the methods that we used seems to have been optimized in any way on data specific to non-extremophiles. Another major problem coming with the diversity of disorder predictions considered for this analysis pertains to the alternative outlier or majority, i.e. should we report what one particular methods sees or should we focus on the consensus of the majority of methods. Again, there seems ample misunderstanding spread in the literature as to this matter. Some methods predicting disorder differ greatly and systematically because they capture different aspects of disorder. Differences between two data sets captured by one method and not by two others may point to the exact reason why that ‘outlier’ method correctly captures a reality missed by the other two. Given the heterogeneity of the phenomenon protein disorder, this seems a very likely interpretation when comparing different methods. In our example, this might indicate that the IUPred prediction that radiation resistant correlates with high disorder might be more helpful than the MD prediction of the opposite trend.

## Conclusions

Extremophiles thrive in environments with extreme conditions such as high salt, exceptionally low or high temperatures and high radiation. We compared organisms through a quite simple criterion, namely the percentage of proteins for which at least one long region of disorder was predicted by 3x4 approaches to predict disorder (three methods, four thresholds). We analyzed protein disorder for several prokaryotic extremophiles and their closest phylogenetic relatives. We found protein disorder to be more reflective of habitat than of the evolutionary relation. This suggested that disordered regions might help crucially in adapting to challenging environments. For example, halophiles appeared to have significantly more protein disorder than their mesophilic relatives suggesting that protein disorder might compensate for the osmotic stress in extremely salty environments. Our data also indicated that the protein disorder differences between habitats depend less on the features of the corresponding taxonomic branch. For instance, both halophilic bacterial and halophilic archaeal proteomes were predicted with more disorder than their taxonomic neighbors. Correspondingly, hyperthermophiles appeared to have less disorder than their mesophilic taxonomic relatives. Finally, we investigated how disordered regions might contribute to environmental adaptation. Comparing the homologues between two extremophiles from cold and heat, we established that more often than expected by chance, disordered regions were found in the cold than in the heat. Largely, it appeared that the level of disorder was rather affected by many small than by few big changes. Overall, protein disorder appeared as a possible building block to bring about evolutionary changes such as the adaptation to different habitats.

## Supporting Information

S1 Fig Processing steps for “completely disordered" approach. (PDF)

S2 Fig Flowchart of statistical steps. (PDF)

S3 Fig Distribution of disorder content in different organisms for %long30. (PDF)

S4 Fig Distribution of disorder content in different organisms for %long50. (PDF)

S5 Fig Distribution of disorder content in different organisms for %long80. (PDF)

S6 Fig Graphical representation for amino acid abundance in different extreme organisms using Z-score. (PDF)

S7 Fig Protein disorder content by environment or phylogeny for %long50. (PDF)

S8 Fig Protein disorder content by environment or phylogeny for %long80. (PDF)

S1 Table List of organisms grouped after environmental conditions. (PDF)

S2 Table Z-score for protein disorder abundance for disorder regions > 30 residues. (PDF)

S3 Table Z-score for protein disorder abundance for disorder regions > 50 residues. (PDF)

S4 Table Z-score for protein disorder abundance for disorder regions > 80 residues. (PDF)

S5 Table Z-score for protein disorder abundance for “completely disordered” proteins. (PDF)

S6 Table Protein disorder abundance for disorder regions > 30 residues. (PDF)

S7 Table Protein disorder abundance for disorder regions > 50 residues. (PDF)

S8 Table Protein disorder abundance for disorder regions > 80 residues. (PDF)

S9 Table Protein disorder abundance for completely disordered proteins. (PDF)

S10 Table Overlap in protein disorder between a hyperthermophile and a mesophile. (PDF)

S11 Table Overlap in protein disorder between a psychrophile and a mesophile. (PDF)

S12 Table Relation protein disorder vs. ordered for homologue proteins in two extreme organisms. (PDF)

S13 Table Amino acid distribution on different groups of extreme organisms. (PDF)

S14 Table List of organisms grouped after taxonomical classification. (PDF)

S15 Table Test of equality of variances and medians of the groups for MD predictions (%long30/50/80). (PDF)

S16 Table Test of equality of variances and medians of the groups for NORSnet predictions (%long30/50/80). (PDF)

S17 Table Test of equality of variances and medians of the groups for IUPred predictions (%long30/50/80). (PDF)

S18 Table Test of equality of variances and medians of the groups for algorithm completely disordered. (PDF)

S19 Table Test of equality of variances and medians of the groups for Z-scores of MD predictions (%long30/50/80). (PDF)

S20 Table Test of equality of variances and medians of the groups for Z-scores of NORSnet predictions (%long30/50/80). (PDF)

S21 Table Test of equality of variances and medians of the groups for Z-scores of IUPred predictions (%long30/50/80). (PDF)

S22 Table Test of equality of variances and medians of the groups for Z-scores of algorithm completely disordered. (PDF)
