# Supplementary material for: Environmental Pressure May Change the Composition Protein Disorder in Prokaryotes
Source: PLoS One. 2015 Aug 7;10(8):e0133990. doi: 10.1371/journal.pone.0133990 (PMC4529154; doi:10.1371/journal.pone.0133990)
Supplement: S1 Fig — (PDF) [file pone.0133990.s001.pdf]

Fig. S1:

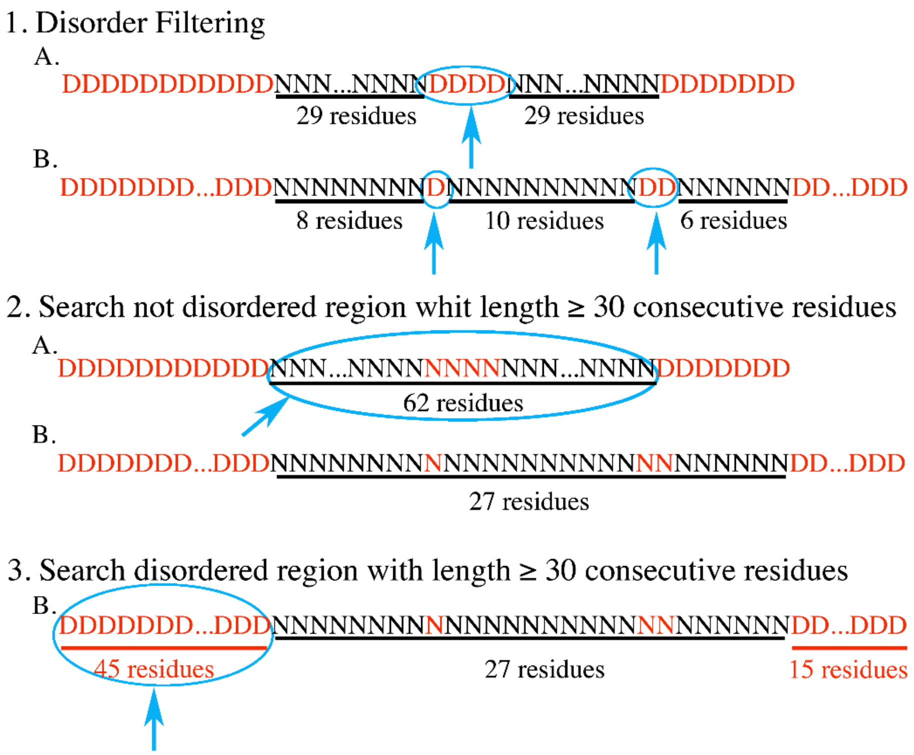

Fig. S1: Processing steps for "completely disordered" approach: This method was considered in order to capture proteins without a single region that we could perceive as a "nucleation site" for adopting regular structure. Operationally, we first removed any prediction of disorder that spanned over fewer than five residues (step 1), next we searched any region without predicted disorder over 30 consecutive residues (step 2). If we found no such region, and if we also found at least one region with ≥30 consecutive residues predicted as disordered, we considered the protein as "completely disordered" (step 3).
