# Supplementary material for: Environmental Pressure May Change the Composition Protein Disorder in Prokaryotes
Source: PLoS One. 2015 Aug 7;10(8):e0133990. doi: 10.1371/journal.pone.0133990 (PMC4529154; doi:10.1371/journal.pone.0133990)
Supplement: S2 Fig — (PDF) [file pone.0133990.s002.pdf]

Fig. S2:

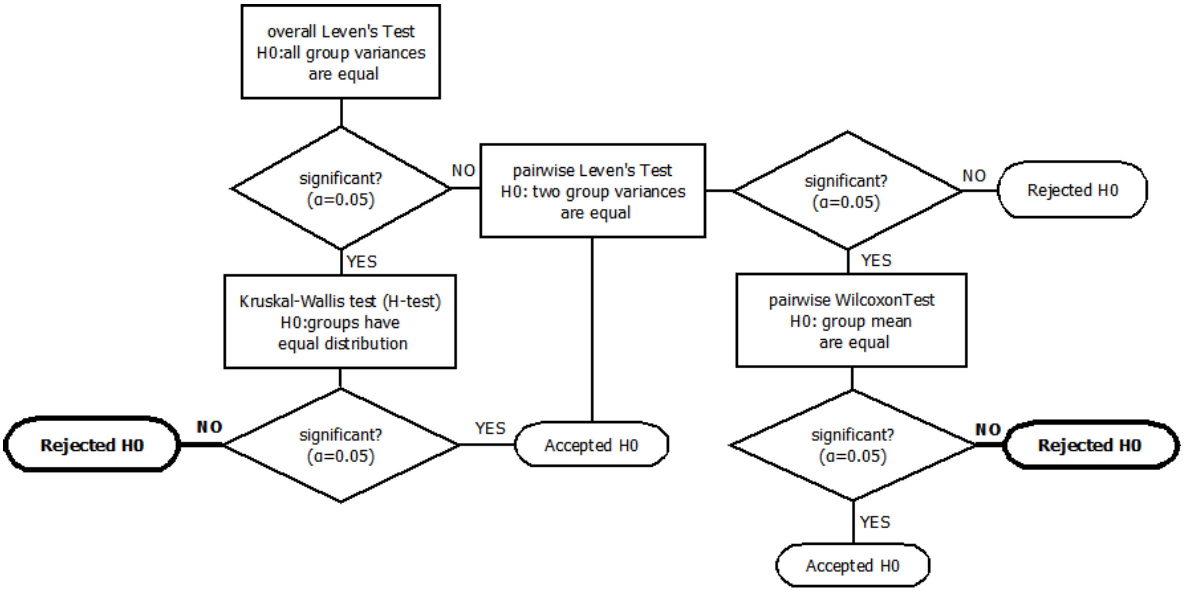

Fig. S2: Graphical representation of the statistical analysis steps. We finalize the statistical test, when the null hypothesis (H0) of the Kruskal-Wallis Test or the pairwise Wilcoxon Test is rejected. That means that the analyzed groups present a significant difference in protein disorder content.
