# Supplementary material for: Environmental Pressure May Change the Composition Protein Disorder in Prokaryotes
Source: PLoS One. 2015 Aug 7;10(8):e0133990. doi: 10.1371/journal.pone.0133990 (PMC4529154; doi:10.1371/journal.pone.0133990)
Supplement: S3 Fig — (PDF) [file pone.0133990.s003.pdf]

Fig. S3:

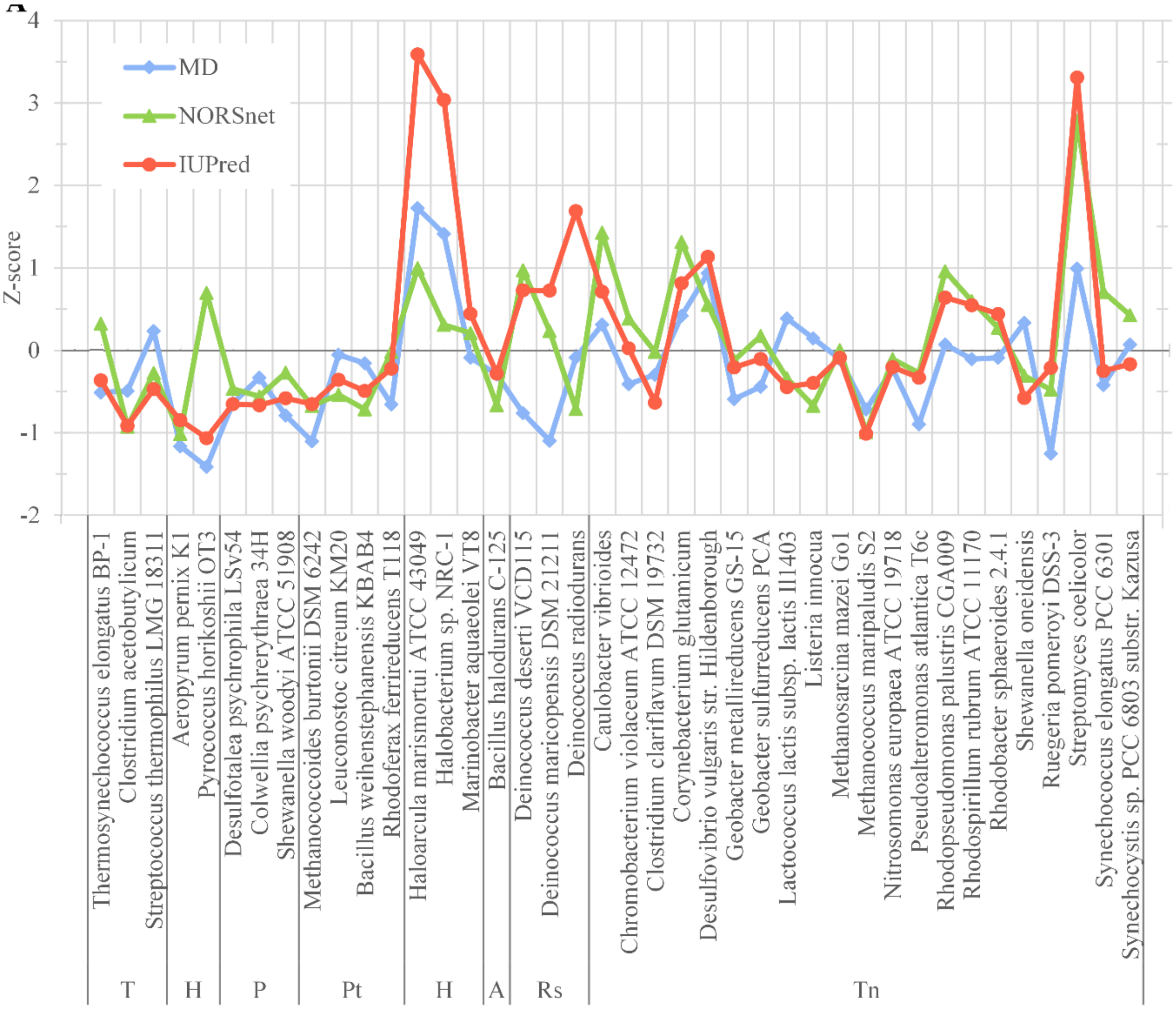

Fig. S3: Distribution of disorder content in different organisms for %long30. Fractions of proteins with long regions of disorder (here ≥30 consecutive residues) were predicted by three prediction methods (MD, NORSnet and IUPred). The raw values are standardized using the Z-scores (Eqn. 1) and the mean and standard deviation (sd) obtained from a population of 1613 prokaryotes calculated for each method (positive: higher than the mean; negative: below the mean; integers +/- N imply N*sd above/below the mean). The taxonomic neighbors section compares the disorder predicted for the closest relatives of the extremophiles.Abbreviations: T, Thermophiles; H, Hyperthermophiles; P, Psychrophiles; Pt, Psychrotolerants; H, Halophiles; A, Ahlkalophla; Rs, Radiation resistents; Tn, Taxonomic neighbors.
