# Supplementary material for: Environmental Pressure May Change the Composition Protein Disorder in Prokaryotes
Source: PLoS One. 2015 Aug 7;10(8):e0133990. doi: 10.1371/journal.pone.0133990 (PMC4529154; doi:10.1371/journal.pone.0133990)
Supplement: S6 Fig — (PDF) [file pone.0133990.s006.pdf]

Fig. S6:

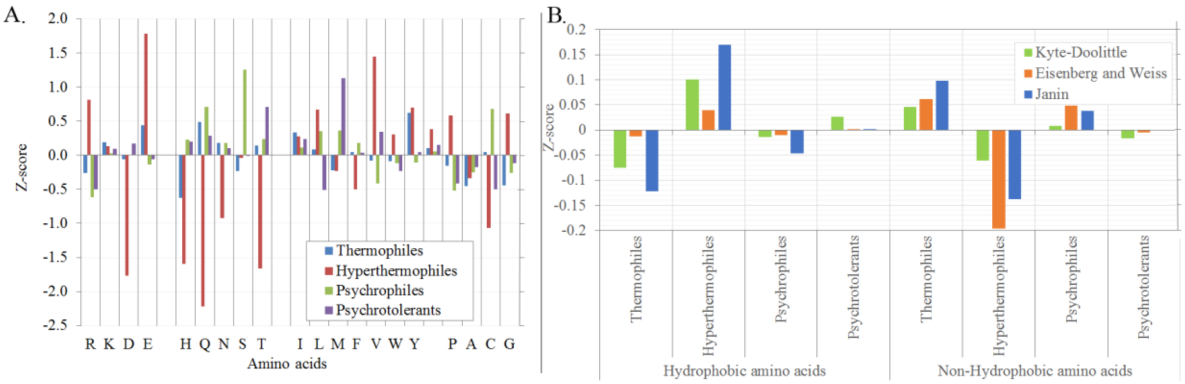

Fig. S6: Graphical representation for amino acid abundance in different extreme organisms using Z-score. Diagram A) containing the single amino acid abundance in whole proteome of hyperthermophiles (red), thermophiles (blue), psychrophiles (green) and psychrotolerants (purple) organisms. Hyperthermophile and thermophile organisms have negative charged Glutamic acid (E, Z-score around 0.4 and 2.0) and aromatic Tyrosine (Y, Z-score around 0.7) and the psychrophile and psychrotolerant lower content o the hydrophobic aliphatic Leucine (L, Z-score between -0.5, -0.3), the charged hydrophobic Arginine (R, Z-score around -1) and the aliphatic turns promoting hydrophilic Proline (P, Z-score from -0.4 to -1.5). We calculate the Z-score using the complete proteome of 1613 prokaryota. To have a general vision about the amino acids content in organisms in terms of hydrophobicity, the amino acids where grouped according to different scales (B). We observe a slight difference on the content of hydrophobic amino acids in thermophiles (lower content) with respect to the other three groups and the depletion of non-hydrophobic amino acids in hyperthermophiles.
