# Supplementary material for: Environmental Pressure May Change the Composition Protein Disorder in Prokaryotes
Source: PLoS One. 2015 Aug 7;10(8):e0133990. doi: 10.1371/journal.pone.0133990 (PMC4529154; doi:10.1371/journal.pone.0133990)
Supplement: S7 Fig — (PDF) [file pone.0133990.s007.pdf]

Fig. S7:

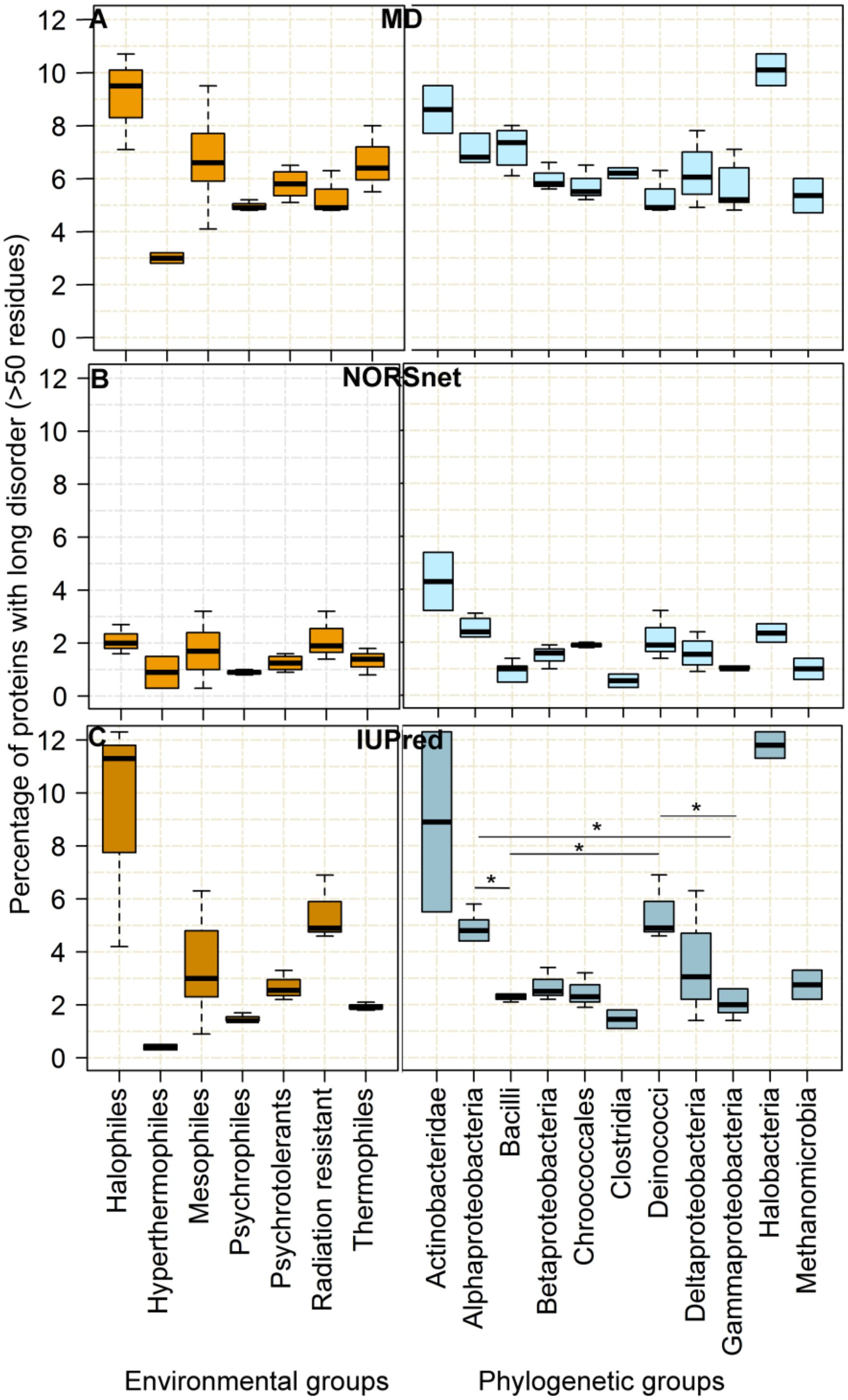

Fig. S7: Protein disorder content by environment or phylogeny for %long50. We represent the protein disorder content for the organisms in the same environment (environmental groups:

A, B and C left side) and those close in terms of evolutionary relation (phylogenetic groups: A, B and C right side). The y-axes give the percentage of proteins with at least one region of ≥50 consecutive residues predicted as disordered by MD (A), NORSnet (B) and IUPred (C). The x-axis on the left side marks the different environmental groups (Table S2) and on the right side marks the studied phylogentic groups (Table S14). The groups which are significant for a paired Wilcoxon Test are marked with * ($P<0.05$) or ** ($P<0.005$).
