# Supplementary material for: Environmental Pressure May Change the Composition Protein Disorder in Prokaryotes
Source: PLoS One. 2015 Aug 7;10(8):e0133990. doi: 10.1371/journal.pone.0133990 (PMC4529154; doi:10.1371/journal.pone.0133990)
Supplement: S8 Fig — (PDF) [file pone.0133990.s008.pdf]

Fig. S8:

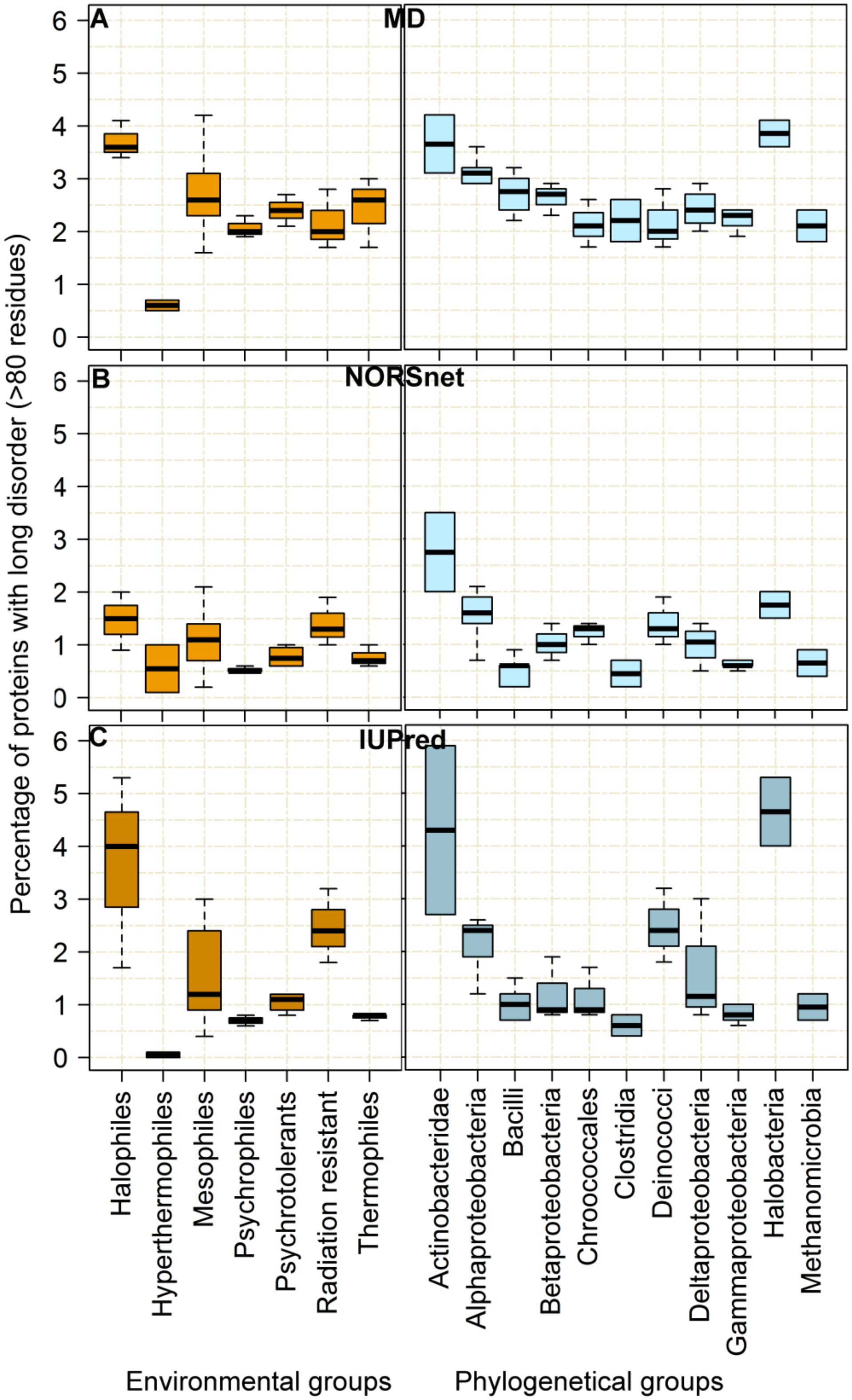
A
MD
B
NORSnet
C
IUPred
Percentage of proteins with long disorder (>80 residues)
0
1
2
3
4
5
6
Halophiles
Hyperthermophiles
Mesophiles
Psychrophiles
Psychrotolerants
Radiation resistant
Thermophiles
Actinobacteridae
Alphaproteobacteria
Bacilli
Betaproteobacteria
Chroococcales
Clostridia
Deinococci
Deltaproteobacteria
Gammaproteobacteria
Halobacteria
Methanomicrobia
Environmental groups
Phylogenetical groups

Fig. S8: Protein disorder content by environment or phylogeny for %long80. We represent the protein disorder content for the organisms in the same environment (environmental groups: A, B and C left side) and those close in terms of evolutionary relation (phylogenetic groups: A, B and C right side). The y-axes give the percentage of proteins with at least one region of ≥80 consecutive residues predicted as disordered by MD (A), NORSnet (B) and IUPred (C). The x-axis on the left side marks the different environmental groups (Table S2) and on the right side marks the studied phylogentic groups (Table S14).
