# Supplementary material for: Environmental Pressure May Change the Composition Protein Disorder in Prokaryotes
Source: PLoS One. 2015 Aug 7;10(8):e0133990. doi: 10.1371/journal.pone.0133990 (PMC4529154; doi:10.1371/journal.pone.0133990)
Supplement: S1 Table — (PDF) [file pone.0133990.s009.pdf]

Table S1: List of organisms grouped after environmental conditions.

Organism groups [a]	Organisms [b]	% Organisms [c]	Proteins [d]	% Proteins [e]
**Thermophiles**	3	6.52	7781	3.45
**Hyperthermophiles**	2	4.35	3746	1.66
**Psychrophiles**	3	10.87	12918	5.73
**Psychrotolerants**	4	4.35	14044	6.23
**Halophiles**	3	6.52	10772	4.78
**Alkaliphiles**	1	2.17	3981	1.77
**Radiation resistant**	3	6.52	9759	4.33
**Total extreme organisms**	19	41.3	63001	27.93
**Mesophiles (Bacteria + Achaea)**	21	45.65	70954	31.46
**Eukaryotes**	6	13.04	91595	40.61
**Total organisms**	46	100.00	225550	100.00

a. Organisms are grouped after the environmental conditions (Thermophiles, organisms with growth temperature optima between 45°C and 80°C; Hyperthermophiles, with optima above 80°C; Pychrophiles, grow best at 15°C or lower; Psychrotolerants, have the capability to wrong at 0°C; Halophiles, organisms adapted to grow best in "salty" solutions, means anywhere from 25% NaCl up to saturation; Alkaliphiles, they pH optimum for grow is above 8, Mesophiles group include the Bacteria and Achaea that are living in "neutral" environmental conditions. Eukaryotes are considered as a different group due their different phylogenetic relationships and protein disorder content respect Achaea and Bacteria.
b. The Organisms field marked the number of organisms that are involved in each group.
c. % Organisms is the fraction of organisms calculated from the Total organisms (46).
d. Proteins N. marks the number of proteins in each group.
e. % Proteins marks the fraction of proteins in each group (as percentage of all 225,500 proteins considered here).
