# Supplementary material for: Environmental Pressure May Change the Composition Protein Disorder in Prokaryotes
Source: PLoS One. 2015 Aug 7;10(8):e0133990. doi: 10.1371/journal.pone.0133990 (PMC4529154; doi:10.1371/journal.pone.0133990)
Supplement: S3 Table — (PDF) [file pone.0133990.s011.pdf]

Table S3: Z-score for protein disorder abundance for disorder regions > 50 residues

Organism [a]	"%long50" [b]		
	MD [c]	IUPred [c]	NORSnet [c]
**Thermophiles**			
Thermosynechococcus elongatus BP-1	-0.5	-0.5	0.2
Clostridium clariflavum DSM 19732	-0.1	-0.6	-0.6
Streptococcus thermophilus LMG 18311	0.3	-0.5	-0.1
**Hyperthermophiles**			
Aeropyrum pernix K1	-1.2	-1.1	-0.9
Pyrococcus horikoshii OT3	-1.4	-1.1	0.2
**Psychrophiles**			
Desulfotalea psychrophila LSv54	-0.7	-0.7	-0.5
Colwellia psychrerythraea 34H	-0.7	-0.7	-0.6
Shewanella woodyi ATCC 51908	-0.6	-0.6	-0.5
**Psychrotolerants**			
Methanococcoides burtonii DSM 6242	-0.7	-0.4	-0.7
Leuconostoc citreum KM20	0.1	-0.2	-0.4
Bacillus weihenstephanensis KBAB4	-0.1	-0.4	-0.5
Rhodoferax ferrireducens T118	-0.4	-0.4	0.0
**Halophiles**			
Haloarcula marismortui ATCC 43049	1.3	3.2	0.8
Halobacterium sp. NRC-1	0.9	2.8	0.3
Marinobacter aquaeolei VT8	0.1	0.3	0.0
**Alkalophile**			
Bacillus halodurans C-125	-0.3	-0.4	-0.8
**Radiation resistant**			
Deinococcus deserti VCD115	-0.7	0.4	0.3
Deinococcus maricopensis DSM 21211	-0.7	0.5	-0.1
Deinococcus radiodurans	-0.1	1.2	1.2
**Taxonomic neighbors (mesophiles)**			
Caulobacter vibrioides	0.3	0.7	1.0
Chromobacterium violaceum ATCC 12472	-0.1	0.0	0.3
Clostridium acetobutylicum	-0.3	-0.8	-1.0
Corynebacterium glutamicum	0.3	0.8	1.2
Desulfovibrio vulgaris str. Hildenborough	0.3	1.1	0.5

Geobacter metallireducens GS-15	-0.2	-0.1	-0.1
Geobacter sulfurreducens PCA	-0.3	-0.1	0.1
Lactococcus lactis subsp. lactis Il1403	0.3	-0.4	-0.3
Listeria innocua	0.1	-0.4	-0.7
Methanosarcina mazei Go1	-0.3	0.0	-0.1
Methanococcus maripaludis S2	-0.7	-0.9	-0.9
Nitrosomonas europaea ATCC 19718	-0.4	-0.4	-0.4
Pseudoalteromonas atlantica T6c	-0.6	-0.3	-0.3
Rhodopseudomonas palustris CGA009	0.2	0.8	1.0
Rhodospirillum rubrum ATCC 11170	-0.1	0.4	0.6
Rhodobacter sphaeroides 2.4.1	0.0	0.4	0.4
Shewanella oneidensis	-0.2	-0.5	-0.4
Ruegeria pomeroyi DSS-3	-0.9	-0.4	-0.5
Streptomyces coelicolor	0.8	3.2	2.7
Synechococcus elongatus PCC 6301	-0.5	-0.4	0.3
Synechocystis sp. PCC 6803 substr. Kazusa	-0.1	0.0	0.2

a. Organism marks the full name of the organism where grey cells correspond to the environments; Taxonomic neighbors correspond to organisms that are related in phylogeny to the extremophiles described in this study. We compiled averages (ave) and standard deviations (sd) over a set of 1,613 complete prokaryotic proteomes taken from UniProt. Eukaryotes are not included due the differences in disorder content (MD_{ave}=6.8%, MD_{sd}=3.0%; $NORsnet_{ave}$=1.6%, $NORsnet_{sd}$=1.4%; $IUPred_{ave}$=3.4% and $IUPred_{sd}$=2.8%).
b. Disorder %long50 refers to the percentage of proteins in a proteome that contains at least one region with ≥50 consecutive residues predicted as disordered.
c. <MD | IUPred | NORSnet> refer to the three prediction methods used, in order to catch the different “flavors” of disorder.
