# Supplementary material for: Environmental Pressure May Change the Composition Protein Disorder in Prokaryotes
Source: PLoS One. 2015 Aug 7;10(8):e0133990. doi: 10.1371/journal.pone.0133990 (PMC4529154; doi:10.1371/journal.pone.0133990)
Supplement: S5 Table — (PDF) [file pone.0133990.s013.pdf]

Table S5: Z-score for protein disorder abundance for "completely disordered" proteins

**Organism** [a]	**"completely disordered"** [b]		
	**MD** [c]	**IUPred** [c]	**NORSnet** [c]
**Thermophiles**			
Thermosynechococcus elongatus BP-1	-0.4	0.0	-0.6
Clostridium clariflavum DSM 19732	0.1	-0.4	-0.7
Streptococcus thermophilus LMG 18311	0.6	-0.3	-0.3
**Hyperthermophiles**			
Aeropyrum pernix K1	-0.6	-0.8	-0.9
Pyrococcus horikoshii OT3	-0.9	5.7	-0.9
Psychrophiles			
Desulfotalea psychrophila LSv54	-0.4	-0.1	-0.5
Colwellia psychrerythraea 34H	0.4	-0.5	-0.6
Shewanella woodyi ATCC 51908	-0.5	-0.2	-0.6
Psychrotolerants			
Methanococcoides burtonii DSM 6242	-0.7	-0.7	-0.7
Leuconostoc citreum KM20	0.3	0.2	-0.5
Bacillus weihenstephanensis KBAB4	0.5	-0.3	0.0
Rhodoferax ferrireducens T118	-0.6	0.0	-0.4
**Halophiles**			
Haloarcula marismortui ATCC 43049	1.9	1.3	4.7
Halobacterium sp. NRC-1	1.8	1.5	4.7
Marinobacter aquaeolei VT8	-0.1	-0.3	-0.1
**Alkalophile**			
Bacillus halodurans C-125	0.3	-0.3	0.2
**Radiation resistant**			
Deinococcus deserti VCD115	-0.7	1.4	1.0
Deinococcus maricopensis DSM 21211	-0.8	0.9	1.0
Deinococcus radiodurans	-0.4	3.0	1.6
**Taxonomic neighbors (mesophiles)**			
Caulobacter vibrioides	0.4	3.9	2.0
Chromobacterium violaceum ATCC 12472	0.1	1.4	0.5
Clostridium acetobutylicum	0.1	-0.5	-0.8
Corynebacterium glutamicum	0.0	0.7	0.5
Desulfovibrio vulgaris str. Hildenborough	1.1	0.6	1.5

Geobacter metallireducens GS-15	-0.3	0.0	-0.5
Geobacter sulfurreducens PCA	0.1	0.5	0.2
Lactococcus lactis subsp. lactis Il1403	0.5	-0.2	-0.4
Listeria innocua	0.4	-0.5	-0.5
Methanosarcina mazei Go1	0.0	-0.1	-0.5
Methanococcus maripaludis S2	-0.3	-0.8	-0.9
Nitrosomonas europaea ATCC 19718	0.1	-0.6	-0.6
Pseudoalteromonas atlantica T6c	-0.7	-0.3	-0.4
Rhodopseudomonas palustris CGA009	-0.2	1.6	0.7
Rhodospirillum rubrum ATCC 11170	-0.3	0.9	0.2
Rhodobacter sphaeroides 2.4.1	0.0	1.5	1.0
Shewanella oneidensis	1.1	-0.6	-0.3
Ruegeria pomeroyi DSS-3	-1.0	-0.3	-0.4
Streptomyces coelicolor	0.0	2.1	2.8
Synechococcus elongatus PCC 6301	-0.8	0.4	-0.6
Synechocystis sp. PCC 6803 substr. Kazusa	0.1	0.1	-0.3

a. Organism marks the full name of the organism where grey cells correspond to the environments; Taxonomic neighbors correspond to organisms that are related in phylogeny to the extremophiles described in this study. We compiled averages (ave) and standard deviations (sd) over a set of 1,613 complete prokaryotic proteomes taken from UniProt. Eukaryotes are not included due the differences in disorder content (MD_{ave}=7.7%, MD_{sd}=3.0%; $NORsnet_{ave}$=0.3%, $NORsnet_{sd}$=0.3%; $IUPred_{ave}$=0.9% and $IUPred_{sd}$=0.8%).
b. Disorder "completely disordered" refers to the percentage of proteins in a proteome that contains proteins with no single region that we could perceive as a "nucleation site" for adopting regular structure (Fig. S1).
c. <MD | IUPred | NORSnet> refer to the three prediction methods used, in order to catch the different "flavors" of disorder.
