# Supplementary material for: Environmental Pressure May Change the Composition Protein Disorder in Prokaryotes
Source: PLoS One. 2015 Aug 7;10(8):e0133990. doi: 10.1371/journal.pone.0133990 (PMC4529154; doi:10.1371/journal.pone.0133990)
Supplement: S6 Table — (PDF) [file pone.0133990.s014.pdf]

Table S6: Protein disorder abundance for disorder regions > 30 residues.

**Organism** [a]	**"%long30"** [b]		
	**MD** [c]	**IUPred** [c]	**NORSnet** [c]
**Thermophiles**			
Thermosynechococcus elongatus BP-1	12 ± 1	5.4 ± 0.9	3.2 ± 0.6
Clostridium clariflavum DSM 19732	13 ± 1	4.0 ± 0.6	1.1 ± 0.3
Streptococcus thermophilus LMG 18311	16 ± 2	5 ± 1	2.0 ± 0.6
**Hyperthermophiles**			
Aeropyrum pernix K1	9 ± 1	2.8 ± 0.7	0.5 ± 0.4
Pyrococcus horikoshii OT3	9 ± 1	1.6 ± 0.5	4.0 ± 0.8
**Psychrophiles**			
Desulfotalea psychrophila LSv54	12 ± 1	3.9 ± 0.6	1.5 ± 0.4
Colwellia psychrerythraea 34H	12.2 ± 0.9	3.8 ± 0.5	1.1 ± 0.3
Shewanella woodyi ATCC 51908	11.3 ± 0.8	4.3 ± 0.5	2.0 ± 0.4
**Psychrotolerants**			
Methanococcoides burtonii DSM 6242	10 ± 1	3.9 ± 0.7	1.2 ± 0.4
Leuconostoc citreum KM20	14 ± 1	6 ± 1	1.4 ± 0.5
Bacillus weihenstephanensis KBAB4	14.0 ± 0.9	4.7 ± 0.5	1.1 ± 0.3
Rhodoferax ferrireducens T118	11.8 ± 0.9	6.2 ± 0.7	2.5 ± 0.4
**Halophiles**			
Haloarcula marismortui ATCC 43049	22 ± 1	27 ± 1	4.6 ± 0.6
Halobacterium sp. NRC-1	21 ± 1	24 ± 2	3.2 ± 0.7
Marinobacter aquaeolei VT8	14 ± 1	9.9 ± 0.8	3.0 ± 0.5
**Alkalophile**			
Bacillus halodurans C-125	13 ± 1	5.9 ± 0.7	1.2 ± 0.3
**Radiation resistant**			
Deinococcus deserti VCD115	11 ± 1	11.4 ± 0.9	3.3 ± 0.6
Deinococcus maricopensis DSM 21211	10 ± 1	11 ± 1	2.5 ± 0.5
Deinococcus radiodurans	14 ± 1	17 ± 1	5.2 ± 0.7
**Taxonomic neighbors (mesophiles)**			

Caulobacter vibrioides	16 ± 1	11 ± 1	4.5 ± 0.6
Chromobacterium violaceum ATCC 12472	12.9 ± 0.9	7.6 ± 0.7	3.0 ± 0.4
Clostridium acetobutylicum	13 ± 1	2.4 ± 0.4	0.6 ± 0.2
Corynebacterium glutamicum	16 ± 1	12 ± 1	5.4 ± 0.7
Desulfovibrio vulgaris str. Hildenborough	19 ± 1	14 ± 1	3.7 ± 0.6
Geobacter metallireducens GS-15	12 ± 1	6.3 ± 0.8	2.3 ± 0.4
Geobacter sulfurreducens PCA	13 ± 1	6.9 ± 0.8	2.9 ± 0.5
Lactococcus lactis subsp. lactis Il1403	16 ± 1	5.0 ± 0.8	1.8 ± 0.5
Listeria innocua	15 ± 1	5.3 ± 0.7	1.2 ± 0.4
Methanosarcina mazei Go1	14 ± 1	7.0 ± 0.8	2.5 ± 0.5
Methanococcus maripaludis S2	12 ± 1	1.9 ± 0.6	0.5 ± 0.3
Nitrosomonas europaea ATCC 19718	14 ± 1	6.3 ± 0.9	2.3 ± 0.6
Pseudoalteromonas atlantica T6c	10.8 ± 0.9	5.6 ± 0.6	2.0 ± 0.4
Rhodopseudomonas palustris CGA009	14.9 ± 0.9	11.0 ± 0.8	4.5 ± 0.6
Rhodospirillum rubrum ATCC 11170	14 ± 1	10.5 ± 0.9	3.7 ± 0.5
Rhodobacter sphaeroides 2.4.1	14 ± 1	9.9 ± 0.9	3.1 ± 0.5
Shewanella oneidensis	16 ± 1	4.3 ± 0.5	1.9 ± 0.4
Ruegeria pomeroyi DSS-3	9.3 ± 0.8	6.3 ± 0.7	1.6 ± 0.4
Streptomyces coelicolor	18.9 ± 0.8	25.8 ± 0.9	8.2 ± 0.6
Synechococcus elongatus PCC 6301	13 ± 1	6.1 ± 0.9	4.0 ± 0.7
Synechocystis sp. PCC 6803 substr. Kazusa	15 ± 1	6.5 ± 0.8	3.4 ± 0.6
**Eukaryotes**			
Arabidopsis thaliana	40.9 ± 0.5	29.8 ± 0.5	31.5 ± 0.5
Caenorhabditis elegans	39.8 ± 0.6	32.3 ± 0.6	28.5 ± 0.5
Dictyostelium discoideum	46.4 ± 0.8	43.8 ± 0.8	28.3 ± 0.7
Drosophila melanogaster	50.7 ± 0.8	45.8 ± 0.8	39.9 ± 0.8
Schizosaccharomyces pombe 972h-	43 ± 1	30 ± 1	29 ± 1
Saccharomyces cerevisiae S288c	46 ± 1	35 ± 1	30 ± 1

a. Organism marks the full name of the organism where grey cells correspond to the environments; Taxonomic neighbors correspond to organisms that are related in phylogeny to the extremophiles described in this study. Eukaryotes picked at random from the set of completely sequenced organisms in UniProt.
b. Disorder %long30 refers to the percentage of proteins in a proteome that contains at least one region with ≥30 consecutive residues predicted as disordered.
c. <MD | IUPred | NORSnet> refer to the three prediction methods used, in order to catch the different “flavors” of disorder.
