# Supplementary material for: Environmental Pressure May Change the Composition Protein Disorder in Prokaryotes
Source: PLoS One. 2015 Aug 7;10(8):e0133990. doi: 10.1371/journal.pone.0133990 (PMC4529154; doi:10.1371/journal.pone.0133990)
Supplement: S9 Table — (PDF) [file pone.0133990.s017.pdf]

Table S9: Protein disorder abundance for completely disordered proteins.

Organism [a]	"Completely disordered" [b]		
	MD [c]	IUPred [c]	NORSnet c
**Thermophiles**			
Thermosynechococcus elongatus BP-1	5.8 ± 0.8	0.4 ± 0.2	0.3 ± 0.2
Clostridium clariflavum DSM 19732	7.2 ± 0.7	0.4 ± 0.2	0.1 ± 0.1
Streptococcus thermophilus LMG 18311	9 ± 1	0.7 ± 0.4	0.2 ± 0.2
**Hyperthermophiles**			
Aeropyrum pernix K1	5 ± 1	0.2 ± 0.2	0
Pyrococcus horikoshii OT3	4.1 ± 0.8	0.2 ± 0.2	2.1 ± 0.6
**Psychrophiles**			
Desulfotalea psychrophila LSv54	5.9 ± 0.7	0.4 ± 0.2	0.2 ± 0.2
Colwellia psychrerythraea 34H	8.2 ± 0.7	0.4 ± 0.2	0.08 ± 0.08
Shewanella woodyi ATCC 51908	5.6 ± 0.6	0.4 ± 0.2	0.2 ± 0.1
**Psychrotolerants**			
Methanococcoides burtonii DSM 6242	5.2 ± 0.9	0.3 ± 0.2	0.04 ± 0.07
Leuconostoc citreum KM20	8 ± 1	0.3 ± 0.2	0.4 ± 0.3
Bacillus weihenstephanensis KBAB4	8.6 ± 0.7	0.8 ± 0.2	0.16 ± 0.09
Rhodoferax ferrireducens T118	5.1 ± 0.6	0.6 ± 0.2	0.3 ± 0.1
**Halophiles**			
Haloarcula marismortui ATCC 43049	12 ± 1	4.1 ± 0.6	0.7 ± 0.2
Halobacterium sp. NRC-1	12 ± 1	4.1 ± 0.8	0.8 ± 0.3
Marinobacter aquaeolei VT8	6.6 ± 0.7	0.8 ± 0.3	0.1 ± 0.1
**Alkalophile**			
Bacillus halodurans C-125	8.1 ± 0.8	1.1 ± 0.3	0.2 ± 0.1
**Radiation resistant**			
Deinococcus deserti VCD115	4.9 ± 0.7	1.5 ± 0.4	0.8 ± 0.3
Deinococcus maricopensis DSM 21211	4.9 ± 0.7	1.7 ± 0.4	0.6 ± 0.3
Deinococcus radiodurans	6.0 ± 0.8	2.0 ± 0.5	1.0 ± 0.3
**Taxonomic neighbors (mesophiles)**			
Caulobacter vibrioides	8.1 ± 0.8	2.4 ± 0.4	1.6 ± 0.4
Chromobacterium violaceum ATCC 12472	7.4 ± 0.7	1.3 ± 0.3	0.7 ± 0.2
Clostridium acetobutylicum	7.7 ± 0.8	0.3 ± 0.2	0.10 ± 0.09
Corynebacterium glutamicum	6.9 ± 0.7	1.2 ± 0.3	0.5 ± 0.2
Desulfovibrio vulgaris str. Hildenborough	10.7 ± 0.9	2.0 ± 0.4	0.5 ± 0.2
Geobacter metallireducens GS-15	6.3 ± 0.8	0.4 ± 0.2	0.3 ± 0.2
Geobacter sulfurreducens PCA	7.4 ± 0.8	1.0 ± 0.3	0.4 ± 0.2
Lactococcus lactis subsp. lactis Il1403	9 ± 1	0.5 ± 0.3	0.2 ± 0.2
Listeria innocua	8.3 ± 0.9	0.4 ± 0.2	0.1 ± 0.1

Methanosarcina mazei Go1	6.9 ± 0.8	0.5 ± 0.2	0.3 ± 0.2
Methanococcus maripaludis S2	6 ± 1	0.2 ± 0.2	0
Nitrosomonas europaea ATCC 19718	6.7 ± 0.9	0.3 ± 0.2	0.1 ± 0.1
Pseudoalteromonas atlantica T6c	4.9 ± 0.6	0.5 ± 0.2	0.2 ± 0.1
Rhodopseudomonas palustris CGA009	6.2 ± 0.6	1.3 ± 0.3	0.8 ± 0.2
Rhodospirillum rubrum ATCC 11170	6.1 ± 0.7	1.0 ± 0.3	0.6 ± 0.2
Rhodobacter sphaeroides 2.4.1	7.0 ± 0.7	1.6 ± 0.3	0.8 ± 0.2
Shewanella oneidensis	10.7 ± 0.8	0.6 ± 0.2	0.09 ± 0.08
Ruegeria pomeroyi DSS-3	4.2 ± 0.6	0.5 ± 0.2	0.8 ± 0.2
Streptomyces coelicolor	7.0 ± 0.5	2.9 ± 0.3	0.9 ± 0.2
Synechococcus elongatus PCC 6301	4.8 ± 0.8	0.4 ± 0.2	0.4 ± 0.2
Synechocystis sp. PCC 6803 substr. Kazusa	7.0 ± 0.8	0.5 ± 0.2	0.3 ± 0.2
**Eukaryotes**			
Arabidopsis thaliana	10.0 ± 0.3	2.3 ± 2	4.9 ± 0.2
Caenorhabditis elegans	10.3 ± 0.4	3.4 ± 0.2	5.1 ± 0.3
Dictyostelium discoideum	11.5 ± 0.5	3.2 ± 0.3	3.9 ± 0.3
Drosophila melanogaster	10.1 ± 0.4	3.2 ± 0.3	7.2 ± 0.4
Schizosaccharomyces pombe 972h-	9.2 ± 0.7	2.2 ± 0.4	3.6 ± 0.5
Saccharomyces cerevisiae S288c	8.9 ± 0.6	1.8 ± 0.3	4.3± 0.5

a. Organism marks the full name of the organism where grey cells correspond to the environments; Taxonomic neighbors correspond to organisms that are related in phylogeny to the extremophiles described in this study. Eukaryotes picked at random from the set of completely sequenced organisms in UniProt.
b. Disorder completely disordered refers to the percentage of proteins in a proteome that contains at least one region with ≥30 consecutive residues predicted as disordered.
c. <MD | IUPred | NORSnet> refer to the three prediction methods used, in order to catch the different “flavors” of disorder.
