# Supplementary material for: Environmental Pressure May Change the Composition Protein Disorder in Prokaryotes
Source: PLoS One. 2015 Aug 7;10(8):e0133990. doi: 10.1371/journal.pone.0133990 (PMC4529154; doi:10.1371/journal.pone.0133990)
Supplement: S10 Table — (PDF) [file pone.0133990.s018.pdf]

Table S10: Overlap in protein disorder between a hyperthermophile and a mesophile.

Similarity [a]	Pyrococcus horikoshii OT3 - Pseudoalteromonas atlantica T6c		Pseudoalteromonas atlantica T6c - Pyrococcus horikoshii OT3	
	Percent of homologues [b]	Disordered homologues [c]	Percent of homologues [b]	Disordered homologues [c]
-20	65.96 ± 0.08	5.75 ± 0.08	80.3 ± 0.2	8.4 ± 0.1
-10	55.5 ± 0.1	4.70 ± 0.04	61.9 ± 0.1	6.7 ± 0.1
0	30.3 ± 0.1	3.1 ± 0.1	26.8 ± 0.1	5.4 ± 0.1
10	10.00 ± 0.07	0.49 ± 0.06	5.97 ± 0.03	3.9 ± 0.2
20	1.38 ± 0.03	0	0.78 ± 0.02	6.1 ± 0.1
30	0.25 ± 0.02	0	0.71 0.005	0

a. Similarity marked the minimum HSSP value defining two proteins to be homologous.
b. Percent of homologues marked the number of proteins in one organism that have at least one homologue in the other organism, over the total number of sequences (i.e 1573 in Pyrococcus horikoshii OT3 and 4423 in Colwellia psychrerythraea 34H).
c. Disordered homologues marked the percent of proteins that have homologues in the other organism and are predicted to contain at least one long unstructured region (> 30 consecutive residues), over the total number of proteins that have homologues in the other organism. Note that 8% of all the Pyrococcus horikoshii OT3 sequences and 10 % of Colwellia psychrerythraea 34H are predicted by MD to have at least one long unstructured region (Table1). Therefore, the proteins that these two organism share tend to be more structured that the average over the whole genome.
