# Supplementary material for: Environmental Pressure May Change the Composition Protein Disorder in Prokaryotes
Source: PLoS One. 2015 Aug 7;10(8):e0133990. doi: 10.1371/journal.pone.0133990 (PMC4529154; doi:10.1371/journal.pone.0133990)
Supplement: S11 Table — (PDF) [file pone.0133990.s019.pdf]

Table S11: Overlap in protein disorder between a psychrophile and a mesophile.

Similarity [a]	Colwellia psychrerythraea 34H - Pseudoalteromonas atlantica T6c		Pseudoalteromonas atlantica T6c - Colwellia psychrerythraea 34H	
	Percent of homologues [b]	Disordered homologues [c]	Percent of homologues [b]	Disordered homologues [c]
-20	83.46 ± 0.06	10.75 ± 0.03	88.7 ± 0.2	9.62 ± 0.05
-10	75.2 ± 0.1	9.25 ± 0.08	84.9 ± 0.2	9.14 ± 0.08
0	62.4 ± 0.1	7.6 ± 0.1	69.9.8 ± 0.2	8.3 ± 0.1
10	45.8 ± 0.1	7.9 ± 0.1	49.2 ± 0.2	7.7 ± 0.2
20	30.11 ± 0.1	8.2 ± 0.3	31.89 ± 0.09	8.4 ± 0.2
30	17.90 ± 0.07	7.7 ± 0.4	19.71 ± 0.07	8.4 ± 0.3

a. Similarity marked the minimum HSSP value defining two proteins to be homologous.
b. Percent of homologues marked the number of proteins in one organism that have at least one homologue in the other organism, over the total number of sequences (i.e 4423 in Colwellia psychrerythraea 34H and 4252 in Pseudoalteromonas atlantica T6c).
c. Disordered homologues marked the percent of proteins that have homologues in the other organism and are predicted to contain at least one long unstructured region (> 30 consecutive residues), over the total number of proteins that have homologues in the other organism. Note that 12 % of all the Colwellia psychrerythraea 34H sequences and 10 % of Pseudoalteromonas atlantica T6c are predicted by MD to have at least one long unstructured region (Table1). Therefore, the proteins that these two organism share tend to be more structured that the average over the whole genome. The both organisms are included in the same phylogenetic order alteromonadales that could explain the high percent of homologues comparing with the obtained from the analysis between Pyrococcus horikoshii OT3 and Pseudoalteromonas atlantica T6c.
