# Supplementary material for: Environmental Pressure May Change the Composition Protein Disorder in Prokaryotes
Source: PLoS One. 2015 Aug 7;10(8):e0133990. doi: 10.1371/journal.pone.0133990 (PMC4529154; doi:10.1371/journal.pone.0133990)
Supplement: S12 Table — (PDF) [file pone.0133990.s020.pdf]

Table S12: Relation protein disorder vs. ordered for homologue proteins in two extreme organisms.

Relation [a]	Colwellia-Pyrococcus [b]	Pyrococcus-Colwellia [b]
Dis-Dis	0	0
Dis-Ord	7	1
Ord -Dis	3	11
Ord- Ord	257	185
Total homologues	267	197
Expected Dis	32	16

a. Relation between the homologue proteins of the studied organisms. Dis-Dis marks the number of homologue proteins that contains at least one long unstructured region (> 30 consecutive residues) in the both organisms (the disordered regions could not overlap), Dis-Ord marks the homologue proteins that are considered as disordered only for the first organism. Ord-Dis marks the proteins that are considered disordered proteins for only the second organism and Ord-Ord marks the homologue proteins that not contain any disordered region in both organisms. The number of expected Dis is the number of disordered proteins that should have at last one disordered region after the prediction of MD for the first considered organisms (i.e 12% of 267 for Colwellia and 8% for Pyoccocus) but the observed number of disordered proteins is less than the expected (i.e 7 for Colwellia and 1 for Pyrococcus). Furthermore they don’t share any disordered region between their homologues.

b. The organisms studied are the pyschrophile Colwellia psychrerythraea 34H (4423 sequences, 12% proteins are predicted to contain at least one long unstructured region > 30 consecutive residues) and the hyperthemophile Pyrococcus horikoshii OT3 (1573 sequences, 8% proteins are predicted to contain at least one long unstructured region > 30 consecutive residues).
