# Supplementary material for: Environmental Pressure May Change the Composition Protein Disorder in Prokaryotes
Source: PLoS One. 2015 Aug 7;10(8):e0133990. doi: 10.1371/journal.pone.0133990 (PMC4529154; doi:10.1371/journal.pone.0133990)
Supplement: S13 Table — (PDF) [file pone.0133990.s021.pdf]

Table S13: Amino acid distribution on different groups of extreme organisms.

AAs[a]	Thermophile[b]	Hyperthermophile[c]	Psychrophile[d]	Psychrotolerant[e]
**R**	-0.3	0.8	-0.6	-0.5
**K**	0.2	0.1	0.0	0.1
**D**	-0.1	-1.8	0.0	0.2
**E**	0.4	1.8	-0.1	-0.1
**H**	-0.6	-1.6	0.2	0.2
**Q**	0.5	-2.2	0.7	0.3
**N**	0.2	-0.9	0.2	0.1
**S**	-0.2	0.0	1.3	0.0
**T**	0.1	-1.7	0.2	0.7
**I**	0.3	0.3	0.1	0.2
**L**	0.1	0.7	0.3	-0.5
**M**	-0.2	-0.2	0.4	1.1
**F**	0.0	-0.5	0.2	0.0
**V**	-0.1	1.4	-0.4	0.3
**W**	-0.1	0.3	-0.1	-0.2
**Y**	0.6	0.7	0.1	0.0
**P**	-0.2	0.6	-0.5	-0.4
**A**	-0.4	-0.3	-0.2	-0.2
**C**	0.0	-1.1	0.7	-0.5
**G**	-0.4	0.6	-0.3	-0.1

a. List of the 20 encoded standard amino acids that are considered in the analysis. The amino acids have been sorted by their biophysical features.

b. Mean amino acid content as percent in the protein sequences of the thermophile organisms (see Table S1).

c. Mean amino acid content as percent in the protein sequences of the hyperthermophile organisms (see Table S1).

d. Mean amino acid content in percent for the psychrophile organisms (see Table S1).

e. Mean amino acid content in percent for the psychrotolerant organisms (see Table S1).
