# Supplementary material for: Environmental Pressure May Change the Composition Protein Disorder in Prokaryotes
Source: PLoS One. 2015 Aug 7;10(8):e0133990. doi: 10.1371/journal.pone.0133990 (PMC4529154; doi:10.1371/journal.pone.0133990)
Supplement: S14 Table — (PDF) [file pone.0133990.s022.pdf]

Table S14: List of organisms grouped after several factors

**Factor**[a]	**Organisms** [b]	**Extreme organisms** [c]
**Phylogeny** [d]		
Actinobacteridae	2*	0
Alphaproteobacteria	5*	0
Bacilli	6*	3 (alkalo, pyschrotol, thermo)*
Betaproteobacteria	3*	1 (pyschrotol)*
Chroococcales	3*	1 (thermo)*
Clostridia	2*	1 (thermo)*
Deinococci	3*	3 (radio res)*
Deltaproteobacteria	4*	1 (psychro)*
Gammaproteobacteria	5*	4 (pyschro,halo, pyschrotol,psychro)*
Halobacteria	2*	2 (halo)*
Methanococci	1	0
Methanomicrobia	2*	1 (psychrotol)*
Thermococci	1	1 (hyperthermo)
Thermoprotei	1	1 (hyperthermo)
**Total**	40 (37*)	19 (17*)
**pH (mean)**[e]		
6.4	1	0 (meso)
6.6	1	1 (halo)
6.8	4*	1 (psychrotol)*
6.9	2*	1(psychrotol)*
7.1	2*	1 (radi)*
7.2	4*	3 (radi, pyschro, thermo)*
7.3	3*	1 (radi)*
7.4	2*	1 (halo)*
7.5	4*	3 (radi, pyschro, thermo)*
7.6	1	1 (pyschro)

7.8	1	0
8	2	1 thermo)*
9.5	1	1 (alkalo)
**Total**	28 (21*)	15 (12*)
**Temperature[e]**		
8	1	1 (pyschro)
10	1	1 (pyschro)
20-30	1	0
23.4	1	0
25	3	2 (psychro, pyschroto)*
25-30	1	0
25-35	3	0*
25-40	2	0*
30	3	1(halo)*
30-37	2	1(radi)*
30-40	1	1(pyschroto)
35	1	0
40	2	1(radi)
40-50	1	1(halo)
42	1	1(halo)
45	1	1(thermo)
55	2	2(thermo)*
80-102	1	1(hyperthermo)
**Total**	28 (17*)	14 (6*)
**Oxygen requeriment[e]**		
Aerobe	10*	7(hyperthermo,radi,halo, pyschrotol)*
Anaerobe	8*	4 (thermo,psychro,psychrotol, halo)*
Facultative	17	6(alkalo,psychroto,psychro, thermo)*
Obligate aerobe	1	0
Obligate anaerobe	3*	1 (hyperthermo)*
**Total**	39 (38*)	18 (18*)
**Phenotipe[e]**		
Alkalophile	1	1 (alkalo)

Antimicrobial activities	1	0
Chemoorganotroph	1	1 (psychrotol)
Fast growing	1	0
Heterotroph	2*	0
Heterotroph, Chemolithoautotroph	1	1 (hyperthermo)
Nitrogen cycle	1	0
Non-Pathogen	5*	2 (psychrotol,thermo)*
Non-Pathogen, Biofilm	1	1(halo)
Proteolytic	1	1(halo)
Pathogen	1	0
Radiation resistant	3*	1 (radi)*
Sulfur cycle	1	1(psychro)
**Total**	20 (10*)	9 (3*)
**Cell shape**[e]		
Coccus-shaped	9*	6 (radi,pyschrotol, hyperthermo, thermo)*
Filament-shaped	1	0
Pleomorphic-shaped	1	1(halo)
Rod-shaped	23*	11(alkalo,pyschrotol, pyschro,thermo,halo)*
Sphere-shaped	3*	1 (hyperthermo)*
Spiral-shaped	1	0
**Total**	38 (35*)	19 (18*)
**Energy source**[e]		
Chemolithoautotroph	1	0
Chemolithotroph	2*	0
Chemoorganotroph	8*	5(thermos, radi,halo)*

Heterotroph	2*	2 (psychro,halo)*
Lithotroph	2*	1 (pyschrotol)*
Lithotroph, Chemolithoautotroph	1	0
Photolithotroph, Photoautotroph	1	0
Photosynthetic	1	0
Photosynthetic, Photoautotrop	3*	1(thermo)*
Phototroph	1	0
**Total**	22 (17*)	9 (9*)
**Habitat[e]**		
Aquatic, Fresh water	3*	0
Aquatic, Fresh water, Antarctic	1	0
Aquatic, Fresh water	1	0
Aquatic, Hot spring, Fresh water	1	1 (thermo)
Aquatic, Hot spring, Marine, Hydrothermal ven	1	1 (hyperthermo)
Aquatic, Marine	2*	2 (halo, psychro)*
Aquatic, Ponds, Soil, Marine, Fresh water	1	0
Aquatic, Sediment, Fresh water	1	0
Aquatic, Soil, Host, Fresh water	1	0
Fresh water	1	0
Host	1	0

Host, Soil, Sludge, Mud, Feces	1	1 (psychrotol)
Marine	1	1 (halo)
Marine, Hydrothermal vent	1	1 (hyperthermo)
Milk	1	1(thermo)
Sea water, Salinewater, Extreme	1	1 (halo)
Sediment	1	1 (psychro)
Sediment, Salt marsh, Fresh water	1	0
Soil	8*	4 (psychrotol,radi)*
Soil, Fresh water	3*	1 (alkalo)
Soil, Normal microflora	1	0
Soil, Sediment	1	0
Wastewater, Sludge	1	1(thermo)
**Total**	36 (15*)	16 (6*)
**Cell arrengement[e]**		
Chains	1	0
Chains, Pairs	1	1(thermo)
Chains, Singles	2*	0
Pairs, Singles	3*	1 (psychrol)*
Singles	10*	1 (psychrol,halo)
**Total**	17 (15*)	3(2*)

a. <Factor> marked all the analyzed factors in the statistical part.
b. <Organisms> marked the number of organisms that are involved in each group. The groups marked with an asterisk are considered for the statistical computation.
c. <Extreme organisms> is the number of extreme organisms in this group. The names of the extreme organism groups are between brackets (Table S1). The groups marked with an asterisk (*) are considered for the next statistical computations. Abbreviations: **alkalo**, alkaliphiles; **thermos**, thermophiles; **hyperthermo**, hyperthermophiles; **pychrotol**, psychrotolerants; **pychro**, psychrophile **halo**, halophiles; **radio res**, radio resistant.
d. <Classes> are the taxonomical organism groups grouped after the phylogenetic classification used by NCBI taxonomy database.
e. Marked the general properties of the organisms (metadata) included by the GOLD database which were containing two or more groups. A group was considered in our analysis if it contained more than two samples: pH, Temperature, Oxygen requirement, Phenotype, Cell shape, Energy source, Habitat, and Cell arrangement.
