# Supplementary material for: Environmental Pressure May Change the Composition Protein Disorder in Prokaryotes
Source: PLoS One. 2015 Aug 7;10(8):e0133990. doi: 10.1371/journal.pone.0133990 (PMC4529154; doi:10.1371/journal.pone.0133990)
Supplement: S17 Table — (PDF) [file pone.0133990.s025.pdf]

Table S17: Test of equality of variances and medians of the groups for IUPred predictions (%long30/50/80).

**Factors** [a]	**IUPred** [b]		
	**%long30** [c]	**%long50** [c]	**%long80** [c]
Phylogenetic	**L(P<0.05*); W(alpha-baci**; alpha-gamma*; baci-beta*; baci-deino*; deino-gamma*)**	**L(P<0.05*); W(alpha-baci*; alpha-gamma*; baci-deino*; deino-gamma*)**	L(P=0.11); K(**P<0.05***)
Environment	L(P=0.44); **K(P<0.005**)**	L(P=0.37); **K(P<0.005**)**	L(P=0.29); **K(P<0.05*)**
Temperature	L(P=0.52); K(P=0.46); W(-)	L(P=0.41); K(P=0.32); W(-)	L(P=0.51); K(P=0.53); W(-)
PH-media	**L(P<0.05*)**; W(-)	L(P=0.08); K(P=0.51); W(-)	L(P=0.25); K(P=0.68); W(-)
Oxygen requirement	**L(P<0.005**); W(aero-oblig*; anaero-oblig*; facult-oblig**)**	**L(P<0.005**); W(aero-oblig*; anaero-oblig*; facult-oblig**)**	**L(P<0.005**); W(aero-oblig*; anaero-oblig*; facult-oblig**)**
Phenotype	**L(P<0.005**);** W(-)	**L(P<0.005**);** W(-)	**L(P<0.005**);** W(-)
Cell shape	L(P=0.70); K(P=0.86); W(-)	L(P=0.88); K(P=0.91); W(-)	L(P=0.94); K(P=0.99); W(-)
Energy source	L(P=0.11); K(P=0.78); W(-)	L(P=0.13); K(P=0.73); W(-)	L(P=0.06); K(P=0.76); W(-)
Habitat	**L(P<0.05*)**; W(-)	L(P=0.08); K(P=0.78); W(-)	L(P=0.09); K(P=0.51); W(-)
Cell arrangement	L(P=0.18); K(P=0.13); W(-)	L(P=0.14); K(P=0.12); W(-)	L(P=0.08); K(P=0.11); W(-)

a. <Factors> are several ambient conditions and organism properties used by the GOLD DB as part of a metagenoma description. We included phylogenetic in our study where the groups are based on the NCBI taxonomy database at level of classes. We considered in the study only the factors having more than 10 values (information about more than 10 organisms) and within the factors only the groups containing more than two samples.

b. <IUPred> marked the predictor used.

c. <%long30/50/80 > marked the threshold for the algorithm "long disorder" to classify a protein as disordered or not.

After accepting the null hypothesis of the Levene's test (L), a value of K is considered statistical significant for a p-value (P) < 0.05 and is marked with an asterisk (*; for P< 0.005 with **) and boldfaced. Otherwise an overall Levene's Test followed by a Wilcoxon test are applied between the groups of each factor (pairwise comparisons). Only the groups considered significant by the both test are remarked between brackets and *. Abbreviations: **L**, Brown–Forsythe Levene's test of equality of variances; **K**, the Kruskal-Wallis rank sum statistic; **W**, Wilcoxon signed-rank test with Bonferroni correction; **P**, p-value; **alpha**, alphaproteobacteria; **beta**, betaproteobacteria; **baci**, bacilli; **gamma**, gammaproteobacteria; **deino**, deinococci; **aero**, aerobe; **oblig**, obligate anaerobe; **anaero**, anaerobe; **facult**, facultative.
