# Supplementary material for: Environmental Pressure May Change the Composition Protein Disorder in Prokaryotes
Source: PLoS One. 2015 Aug 7;10(8):e0133990. doi: 10.1371/journal.pone.0133990 (PMC4529154; doi:10.1371/journal.pone.0133990)
Supplement: S18 Table — (PDF) [file pone.0133990.s026.pdf]

Table S18: Test of equality of variances and medians of the groups for algorithm completely disordered.

Factors [a]	Completely disordered [b]		
	**MD [c]**	**IUPred [c]**	**NORSnet [c]**
Phylogenetic	L(P=0.53); K(P=0.25); **W(alpha-baci*; baci-beta*; baci-deino*)**	L(P=0.13); **K(P<0.05*)**	L(P=0.18); **K(P<0.05*)**
Environment	L(P=0.53); K(P=0.24); **W(meso-radi*)**	L(P=0.22); **K(P<0.05*)**	**L(P<0.005*)**; W(-)
Temperature	L(P=0.12); K(P=0.46); W(-)	L(P=0.27); K(P=0.53); W(-)	**L(P<0.05*)**; W(-)
PH-media	**L(P<0.05*)**; W(-)	**L(P<0.005**)**; W(-)	L(P=0.90); K(P=0.97); W(-)
Oxygen requirement	L(P=0.79); K(P=0.50); W(-)	**L(P<0.005**); W(aero-oblig*; anaero-oblig*)**	**L(P<0.05*);** W(-)
Phenotype	**L(P<0.005**);** W(-)	L(P=0.71); K(P=0.09); W(-)	L(P=0.51); K(P=0.09); W(-)
Cell shape	L(P=0.13); K(P=0.90); W(-)	L(P=0.80); K(P=0.62); W(-)	L(P=0.18); K(P=0.71); W(-)
Energy source	L(P=0.66); K(P=0.36); W(-)	L(P=0.33); K(P=0.41); W(-)	L(P=0.08); K(P=0.49); W(-)
Habitat	**L(P<0.05*)**; W(-)	**L(P<0.005*)**; W(-)	L(P=0.11); K(P=0.86); W(-)
Cell arrangement	L(P=0.77); K(P=0.88); W(-)	L(P=0.44); K(P=0.32); W(-)	L(P=0.07); K(P=0.14); W(-)

a. <Factors> are several ambient conditions and organism properties used by the GOLD DB as part of a metagenoma description. We included phylogenetic in our study where the groups are based on the NCBI taxonomy database at level of classes. We considered in

the study only the factors having more than 10 values (information about more than 10 organisms) and within the factors only the groups containing more than two samples.

b. <Completely disordered> marked the algorithm to consider a protein to be disordered.

c. <MD | IUPred | NORSnet> refer to the three prediction methods used, in order to catch the different “flavors” of disorder.

After accepting the null hypothesis of the Levene’s test (L), a value of K is considered statistical significant for a p-value (P) < 0.05 and is marked with an asterisk (*; for P> 0.005 with **) and boldfaced. Otherwise an overall Levene’s Test followed by a Wilcoxon test are applied between the groups of each factor (pairwise comparisons). Only the groups considered significant by the both test are remarked between brackets and *. Abbreviations: **L**, Brown–Forsythe Levene’s test of equality of variances; **K**, the Kruskal-Wallis rank sum statistic; **W**, Wilcoxon signed-rank test with Bonferroni correction; **P**, p-value; **alpha**, alphaproteobacteria; **beta**, betaproteobacteria; **baci**, bacilli; **deino**, deinococci; **meso**, mesophile; **radi**, radiation resistant; **aero**, aerobe; **oblig**, obligate anaerobe; **anaero**, anaerobe.
